# Serine synthesis controls mitochondrial biogenesis in macrophages

**DOI:** 10.1126/sciadv.adn2867

**Published:** 2024-05-17

**Authors:** Chuanlong Wang, Muyang Zhao, Peng Bin, Yuyi Ye, Qingyi Chen, Zhiru Tang, Wenkai Ren

**Affiliations:** ^1^ State Key Laboratory of Swine and Poultry Breeding Industry, College of Animal Science, South China Agricultural University, Guangzhou 510642, China.; ^2^ Animal Nutrition and Bio-feed, College of Animal Science and Technology, Southwest University, Chongqing 400715, China.

## Abstract

Mitochondrial dysfunction is the pivotal driving factor of multiple inflammatory diseases, and targeting mitochondrial biogenesis represents an efficacious approach to ameliorate such dysfunction in inflammatory diseases. Here, we demonstrated that phosphoglycerate dehydrogenase (PHGDH) deficiency promotes mitochondrial biogenesis in inflammatory macrophages. Mechanistically, PHGDH deficiency boosts mitochondrial reactive oxygen species (mtROS) by suppressing cytoplasmic glutathione synthesis. mtROS provokes hypoxia-inducible factor–1α signaling to direct nuclear specificity protein 1 and nuclear respiratory factor 1 transcription. Moreover, myeloid *Phgdh* deficiency reverses diet-induced obesity. Collectively, this study reveals that a mechanism involving de novo serine synthesis orchestrates mitochondrial biogenesis via mitochondrial-to-nuclear communication, and provides a potential therapeutic target for tackling inflammatory diseases and mitochondria-mediated diseases.

## Teaser

De novo serine synthesis controls mitochondrial biogenesis via mitochondrial-to-nuclear communication in macrophages.

## INTRODUCTION

Immune cells play an important role in the occurrence and development of inflammatory diseases (e.g., obesity, type 2 diabetes, and colitis) (*1*, *2*). Mitochondria are important organelles for driving proliferation, differentiation, activation, and polarization of immune cells (e.g., T cells and macrophages) (*3*, *4*). Recent studies have shown that damage of mitochondrial biogenesis and structure promotes inflammatory macrophage responses (*5*–*7*). For example, impaired mitochondrial biogenesis promotes macrophage infiltration and tissue inflammation (*5*). Increasing mitochondrial biogenesis or restoring mitochondrial integrity alleviates macrophage-mediated inflammatory responses by down-regulation of interleukin-1β (IL-1β) and tumor necrosis factor–α (TNF-α) secretion (*8*). Notably, increasing mitochondrial biogenesis is a beneficial treatment for macrophage-mediated inflammatory diseases (e.g., obesity and pneumonia) (*8*–*11*). Those evidence indicate that mitochondrial biogenesis mediates inflammatory macrophage polarization and is associated with inflammatory diseases. However, how to regulate mitochondrial biogenesis in inflammatory macrophages needs further study.

Metabolic reprogramming is an important characteristic for inflammatory macrophage polarization (*12*, *13*). Recent studies have shown that serine metabolism is related to inflammatory macrophage polarization (*14*). Phosphoglycerate dehydrogenase (PHGDH) is the first rate-limiting enzyme in the de novo serine synthesis (*15*). Previous studies have shown that PHGDH is an important target for regulating macrophage polarization (*16*, *17*). PHGDH deficiency enhances mitochondrial number in endothelial cells (*18*). These results indicate that de novo serine synthesis may have a wider and unknown physiological function on macrophage polarization through regulating mitochondrial number or function. However, the underlying mechanism by which de novo serine synthesis regulates mitochondrial function or number in macrophages remains to be uncovered.

In our study, we found that PHGDH deficiency promotes mitochondrial biogenesis in inflammatory macrophages. Mechanistically, PHGDH deficiency suppresses cytoplasmic glutathione (GSH) synthesis but enhances mitochondrial reactive oxygen species (mtROS), which triggers hypoxia-inducible factor–1α (HIF-1α) signaling to regulate nuclear specificity protein 1 (SP1) and nuclear respiratory factor 1 (NRF1) expression, resulting in increased mitochondrial biogenesis. Moreover, myeloid *Phgdh* deficiency reverses diet-induced obesity. Together, our study links de novo serine synthesis with mitochondrial biogenesis in inflammatory macrophages, which provides potential therapeutic targets for treatment of inflammatory diseases.

## RESULTS

### PHGDH inhibition promotes mitochondrial biogenesis

**Fig. 1.**
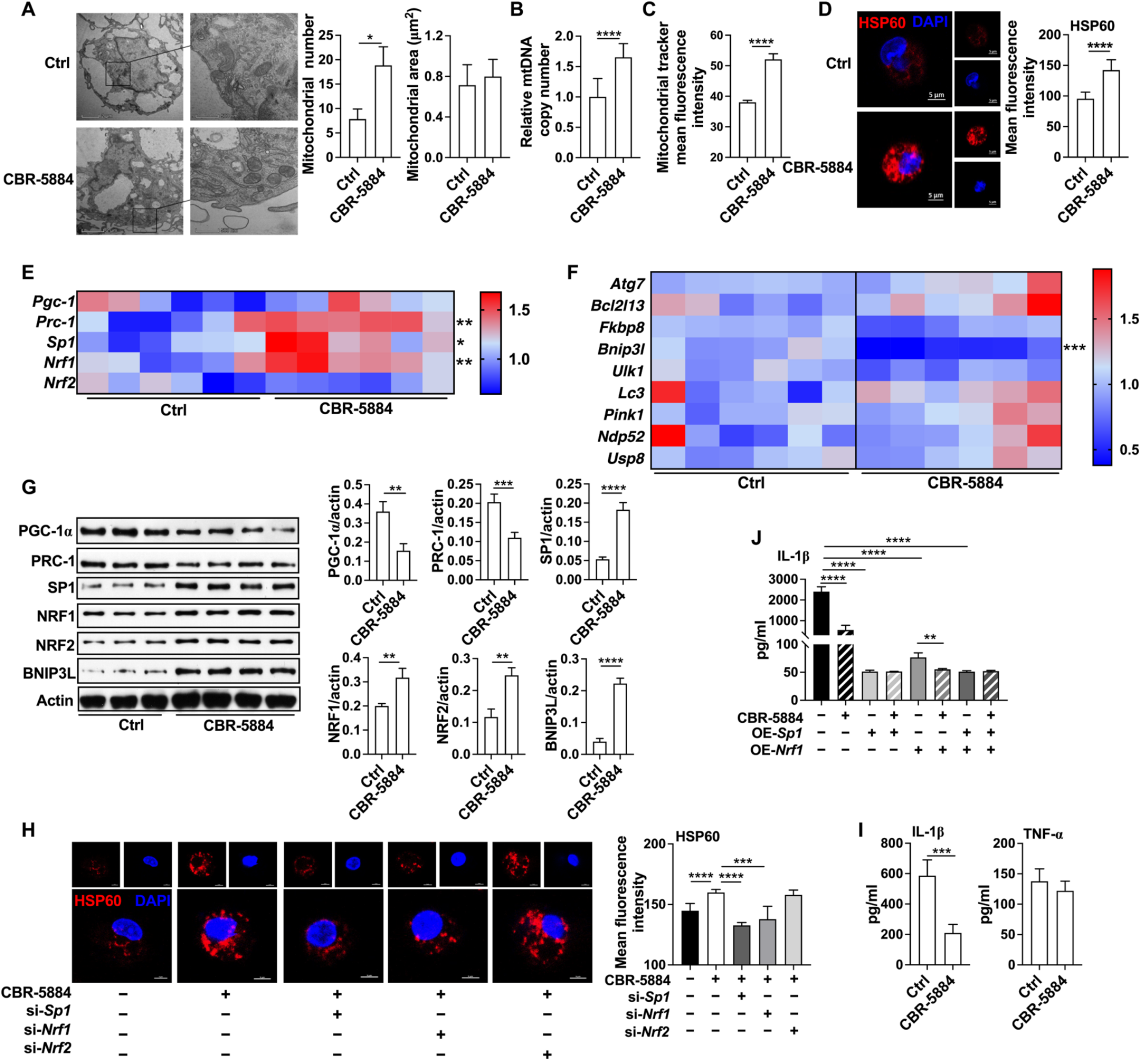
PHGDH inhibition promotes mitochondrial biogenesis. (**A**) Transmission electron microscopy (TEM) observation of mitochondrial number and area in inflammatory macrophages treated with or without CBR-5884 (30 μM) (*n* = 6). Scale bar, 2 μm. (**B** and **C**) The mitochondrial tracker mean fluorescence intensity and mtDNA copy number in inflammatory macrophages treated with or without CBR-5884 (*n* = 9). (**D**) Confocal microscopy of HSP60 (red) in inflammatory macrophages treated with or without CBR-5884 (*n* = 6 or 8). Scale bar, 5 μm. (**E** and **F**) The mRNA expression of *Pgc-1*, *Prc-1*, *Sp1*, *Nrf1*, *Nrf2*, *Atg7*, *Bcl2l13*, *Fkbp8*, *Bnip3l*, *Ulk1*, *Lc3*, *Pink1*, *Ndp52*, and *Usp8* in inflammatory macrophages treated with or without CBR-5884 (*n* = 6). (**G**) Protein abundance of PGC-1α, PRC-1, SP1, NRF1, NRF2, and BNIP3L in inflammatory macrophages treated with or without CBR-5884 (*n* = 3 or 4). (**H**) Confocal microscopy of HSP60 in inflammatory macrophages treated as indicated (*n* = 6). Scale bar, 5 μm. (**I** and **J**) The secretion of IL-1β and TNF-α in inflammatory macrophages treated as indicated (*n* = 4). The treatment time of all experiments was 12 hours. Data were analyzed by unpaired *t* test [(A) to (I)]. (A) (right), (B) to (G), and (I) and (J) are represented as means ± SD; (A) (left) and (H) are represented as means ± SEM. *P < 0.05, **P < 0.01, ***P < 0.001, and ****P < 0.0001.

Although PHGDH is associated with mitochondrial mass (*18*), it is still unclear whether and how PHGDH regulates mitochondrial mass in macrophages. To address this question, we first analyzed the mitochondrial number and area in inflammatory macrophages treated with CBR-5884 (a PHGDH selective inhibitor) (*17*). CBR-5884 increased mitochondrial number, while it had no effect on mitochondrial area in peritoneal macrophages (PEMs) (Fig. 1A), which is consistent with our previous observation with NCT-503 (another PHGDH inhibitor) (*19*). This finding was further supported by analysis of mitochondrial DNA (mtDNA) copy number (Fig. 1B), mitochondrial tracker staining (Fig. 1C), and heat shock protein 60 staining (HSP60, reflecting mitochondrial number) (*20*) (Fig. 1D) with immunofluorescence analysis. However, CBR-5884 had no effect on mitochondrial number in quiescent macrophages (fig. S1A). CBR-5884 also increased mitochondrial number in inflammatory immortalized mouse macrophage ANA-1 (fig. S1B).

Mitochondrial number is regulated by mitochondrial dynamics, mitochondrial biogenesis and mitophagy (*21*–*23*). However, our previous study has demonstrated that PHGDH inhibition suppressed the abundance of dynamin-related protein 1 (associated with higher number of mitochondria) (*19*), suggesting that PHGDH blockage enhances mitochondrial number through mitochondrial biogenesis or mitophagy. Peroxisome proliferator–activated receptor gamma coactivator–1 (PGC-1) and Polycomb repressive complex–1 (PRC-1) interacts with SP1, NRF1, or NRF2 to regulate mitochondrial biogenesis by affecting expression of certain nucleus-encoded mitochondrial genes (*24*). Thus, we analyzed mRNA expression of these key transcription factors associated with mitochondrial biogenesis. CBR-5884 promoted mRNA expression of *Sp1* and *Nrf1* in PEMs or ANA-1 cells (Fig. 1E and fig. S1C). Mitophagy is promoted via specific mitochondrial outer-membrane receptors [e.g., BCL-2 19-kDa interacting protein 3 (BNIP3), BNIP3-like (BNIP3L/NIX), and FUN14 domain containing 1 (FUNDC1)] or ubiquitin molecules conjugated to a series of autophagy adaptors [e.g., nuclear dot protein (NDP52)] on the mitochondrial surface (*25*). We next analyzed mRNA expression of these key factors associated with mitophagy. The results showed that CBR-5884 had no effect on mRNA expression of these factors, except *Bnip3l* in PEMs (Fig. 1F). Among these changed factors, CBR-5884-induced increase of SP1 and NRF1 were also validated from protein level (Fig. 1G).

Thus, we initially hypothesized that PHGDH inhibition promotes mitochondrial biogenesis via SP1 or NRF1. We then silenced SP1, NRF1, and even NRF2 using small interfering RNAs (siRNAs) (fig. S1D). *Sp1* or *Nrf1* silencing, but not *Nrf2* silencing, blocked PHGDH inhibition–induced mitochondrial number in inflammatory macrophages (Fig. 1H). Mitochondrial translation factor A (TFAM), TFB1M and TFB2M are essential for replication and translation of mtDNA (*26*, *27*). Consistently, CBR-5884 promoted mRNA expression of *Tfam*, *Tfb1m*, and *Tfb2m*, while *Sp1* or *Nrf1* silencing blocked *Tfam* mRNA expression in inflammatory macrophages (fig. S1E). The results from chromatin immunoprecipitation (ChIP)–quantitative polymerase chain reaction (qPCR) also showed that CBR-5884 promoted SP1 and NRF1 recruitment to *Tfam* promoter region (fig. S1F). To further confirm the intrinsic function of SP1 or NRF1 in mitochondrial biogenesis, we overexpressed the *Sp1* or *Nrf1* in inflammatory macrophages (fig. S1, G, I, and J). Notably, *Sp1* or *Nrf1* overexpression promoted the mRNA expression of *Tfam* and *Tfb2m* (fig. S1H) and enhanced mitochondrial number (fig. S1, I and J). Together, those findings suggest that PHGDH inhibition promotes mitochondrial biogenesis via SP1/NRF1-TFAM signaling.

Mitochondrial biosynthesis mediates the secretion of inflammatory factors in inflammatory macrophages (*8*). Similar to our previous study (*19*), PHGDH inhibitor suppressed IL-1β secretion while having no effect on TNF-α secretion from inflammatory macrophages (Fig. 1I). To explore the roles of SP1/NRF1-mediated mitochondrial biosynthesis in PHGDH-mediated IL-1β secretion from inflammatory macrophages, we overexpressed the *Sp1* or *Nrf1* to enhance mitochondrial biosynthesis. Intriguingly, *Sp1* or *Nrf1* overexpression reduced IL-1β secretion, and PHGDH blockage failed to limit IL-1β secretion from inflammatory macrophages in the presence of *Sp1* or both *Sp1* and *Nrf1* overexpression (Fig. 1J). Collectively, PHGDH inhibition limits IL-1β secretion from inflammatory macrophages associated with mitochondrial biosynthesis.

### PHGDH inhibition enhances mitochondrial biogenesis through mtROS

**Fig. 2.**
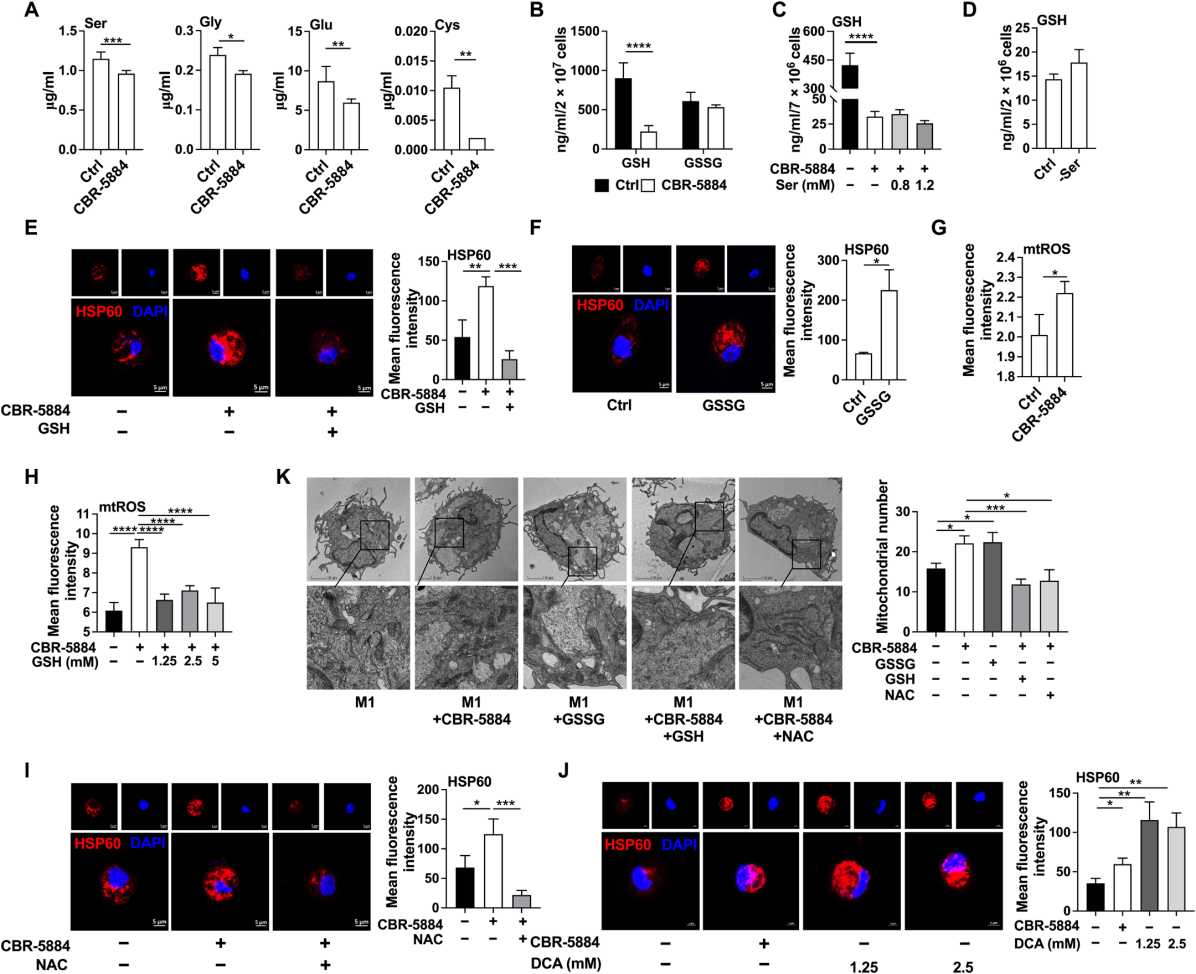
PHGDH inhibition enhances mitochondrial biogenesis through mtROS. (**A** to **D**) The glutamic acid, serine, glycine, cysteine, GSH, and GSSG level in inflammatory macrophages treated as indicated (*n* = 4 or 6). (**E** and **F**) Confocal microscopy of HSP60 (red) in inflammatory macrophages treated as indicated (*n* = 4 or 5) (GSH, 1.25 mM). Scale bar, 5 μm. (**G** and **H**) The mtROS level in inflammatory macrophages treated as indicated (*n* = 3 to 12). (**I** and **J**) Confocal microscopy of HSP60 (red) in inflammatory macrophages treated as indicated (*n* = 4 to 8). Scale bar, 5 μm. (**K**) TEM observation of mitochondrial number in inflammatory macrophages treated as indicated (*n* = 8 to 12) (GSSG and NAC, 1.25 mM). Scale bar, 2 μm. The treatment time of all experiments was 12 hours. Data were analyzed by unpaired *t* test [(A) to (K)]. (A) to (C), (E), and (G) to (I) are represented as means ± SD; (D), (F), and (J) and (K) are represented as means ± SEM. *P < 0.05, **P < 0.01, ***P < 0.001, and ****P < 0.0001.

Subsequently, we explored the underlying mechanism by which PHGDH mediates mitochondrial biogenesis in inflammatory macrophages. Serine synthesis is coupled with glutamic acid and glycine metabolism driving production of GSH (*28*). Consistently, CBR-5884 reduced intracellular levels of serine, glutamic acid, glycine, cysteine, and GSH (Fig. 2, A and B, and fig. S2A). A previous well-designed study has shown that exogenous serine-derived carbons and glycine-derived carbons only accounts for 20 and 10% of GSH in inflammatory macrophages, respectively (*29*), suggesting the production of GSH in inflammatory macrophages is largely dependent on de novo serine synthesis rather than exogenous serine. Notably, exogenous serine supplementation failed to rescue the GSH level in inflammatory macrophages with PHGDH inhibition (Fig. 2C and fig. S2B), and exogenous serine deprivation had no effect on intracellular GSH in inflammatory macrophages (Fig. 2D and fig. S2, C and D). Therefore, inflammatory macrophages produce GSH largely dependent on de novo serine synthesis. To further confirm the subcellular localization of GSH deficiency, we analyzed GSH in the mitochondria or cytoplasm without mitochondria in inflammatory macrophages. CBR-5884 decreased GSH levels in the cytoplasm (fig. S2, E and F). To determine whether PHGDH regulates mitochondrial number via GSH in inflammatory macrophages, we supplemented GSH to inflammatory macrophages (fig. S2G) and found that GSH supplementation decreased mitochondrial number in inflammatory macrophages with PHGDH blockage (Fig. 2, E and K). Thus, PHGDH inhibition mediates mitochondrial biogenesis by limiting GSH synthesis.

To reduce mtROS, GSH is oxidized by glutathione peroxidase to glutathione disulfide (GSSG). GSSG is converted back to GSH by glutathione reductase and cofactor nicotinamide adenine dinucleotide phosphate (*30*). Thus, the balance of GSH/GSSG is an important member of cellular antioxidative system and indispensable for scavenging mtROS (*31*). Thus, we hypothesized that PHGDH inhibition promotes mitochondrial biogenesis via GSH-mtROS signaling. Notably, GSSG supplementation increased mtROS production (fig. S2H) and mitochondrial number in inflammatory macrophages (Fig. 2, F and K). Notably, PHGDH inhibition promoted mtROS production in inflammatory macrophages (Fig. 2G). We then confirmed that PHGDH inhibition promoted mtROS production by blocking GSH synthesis as GSH supplementation (fig. S2G) blocked PHGDH inhibition–induced mtROS production (Fig. 2H). To determine whether PHGDH regulates mitochondrial biogenesis through mtROS, we supplemented *N*-acetylcysteine (NAC, a precursor of GSH to clear mtROS) to inflammatory macrophages (fig. S2I) and found that NAC inhibited mitochondrial number in inflammatory macrophages with PHGDH blockage (Fig. 2, K and I). Furthermore, we analyzed mitochondrial number in inflammatory macrophages treated with sodium dichloroacetate (DCA, a mtROS specific activator) (fig. S2J) (*32*). Like PHGDH inhibitor, DCA enhanced mitochondrial number in inflammatory macrophages (Fig. 2J). These results suggest that PHGDH inhibition enhances mitochondrial biogenesis through mtROS.

### mtROS promotes mitochondrial biogenesis via SP1/NRF1

**Fig. 3.**
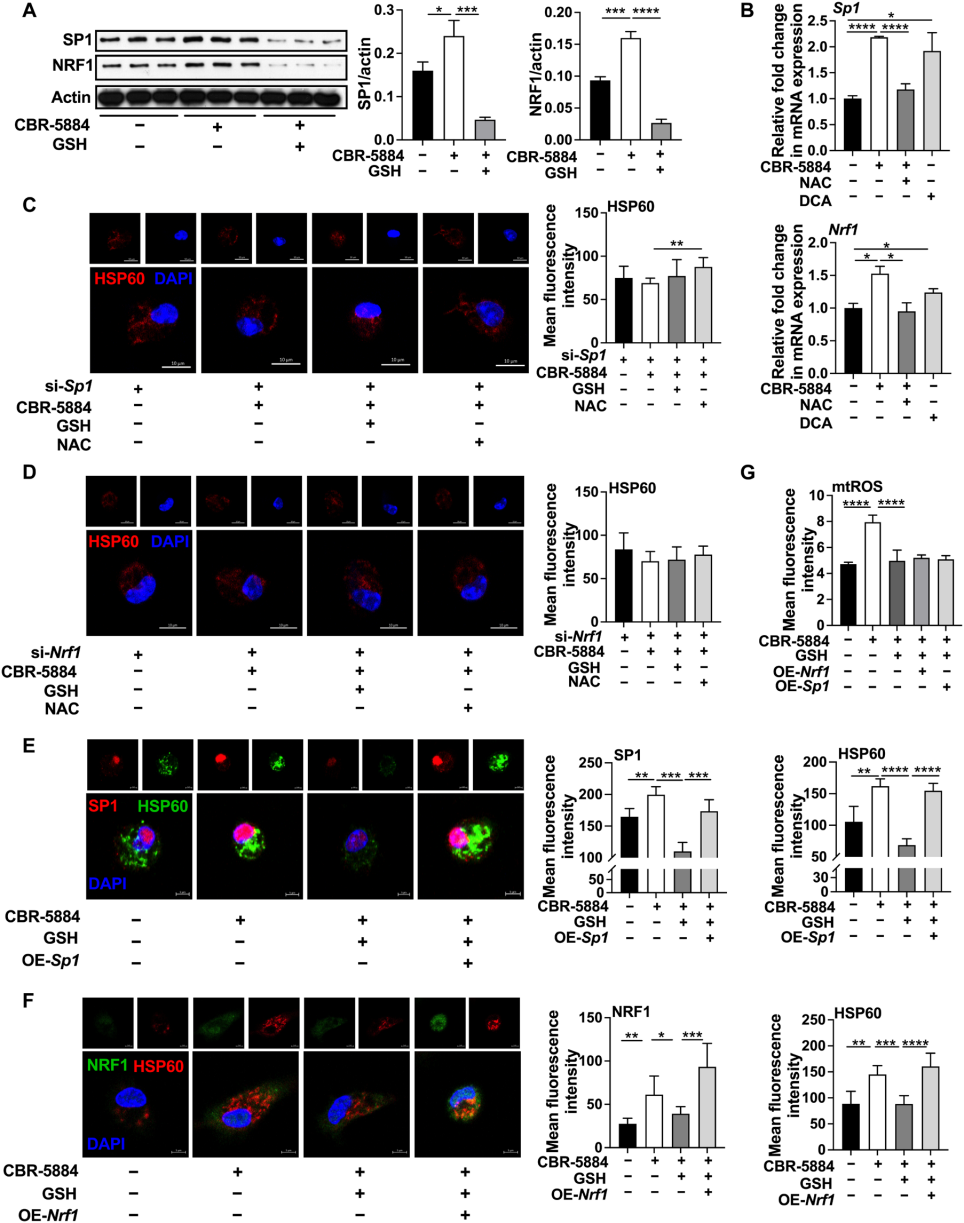
mtROS promotes mitochondrial biogenesis via SP1/NRF1 signaling. (**A**) Protein abundance of SP1 and NRF1 in inflammatory macrophages treated as indicated (*n* = 3). (**B**) Relative mRNA expression of *Sp1* and *Nrf1* in inflammatory macrophages treated as indicated (*n* = 4 to 5). (**C** and **D**) Confocal microscopy of HSP60 in inflammatory macrophages treated as indicated (*n* = 4 to 10). Scale bar, 10 μm. (**E** and **F**) Confocal microscopy of SP1, NRF1, and HSP60 in inflammatory macrophages treated as indicated (*n* = 4 to 8). Scale bar, 5 μm. (G) The mtROS level in inflammatory macrophages treated as indicated (*n* = 7 to 12). The treatment time of all experiments was 12 hours. Data were analyzed by unpaired *t* test [(A) to (G)]. (A) and (C) to (G) are represented as means ± SD. (B) are represented as means ± SEM. *P < 0.05, **P < 0.01, ***P < 0.001, and ****P < 0.0001.

We then asked whether PHGDH inhibition promotes SP1 and NRF1 expression through mtROS. We first supplemented GSH (fig. S2G) to inflammatory macrophages and demonstrated that GSH blocked mRNA expression and protein abundance of SP1 and NRF1 in inflammatory macrophages with PHGDH blockage (Fig. 3A and fig. S3A). In addition, GSSG promoted protein expression of SP1 and NRF1 in inflammatory macrophages (fig. S3B). Consistently, DCA promoted SP1 and NRF1 mRNA expression in inflammatory macrophages, while NAC down-regulated mRNA expression of SP1 and NRF1 in inflammatory macrophages with PHGDH blockage (Fig. 3B).

To demonstrate the critical importance of SP1 or NRF1 in mtROS-induced mitochondrial biogenesis in inflammatory macrophages, we blocked SP1 or NRF1 using siRNAs. After SP1 or NRF1 silence, PHGDH inhibitor failed to enhance mitochondrial number (Fig. 3, C and D). GSH or NAC failed to inhibit mitochondrial number in inflammatory macrophages with PHGDH inhibition (Fig. 3, C and D), and GSSG also failed to change mitochondrial biogenesis (fig. S3, C and D). In addition, *Sp1* or *Nrf1* silencing inhibited mitochondrial number enhanced by GSSG supplementation (fig. S3E). Furthermore, *Sp1* or *Nrf1* overexpression rescued mitochondrial biogenesis in inflammatory macrophages treated with CBR-5884 and GSH (Fig. 3, E and F), while it had little effect on mtROS production (Fig. 3G), indicating that SP1/NRF1 is the downstream of in mtROS. Together, these results suggest that mtROS promotes mitochondrial biogenesis in inflammatory macrophages via SP1/NRF1.

### PHGDH inhibition enhances mitochondrial biogenesis through HIF-1α

**Fig. 4.**
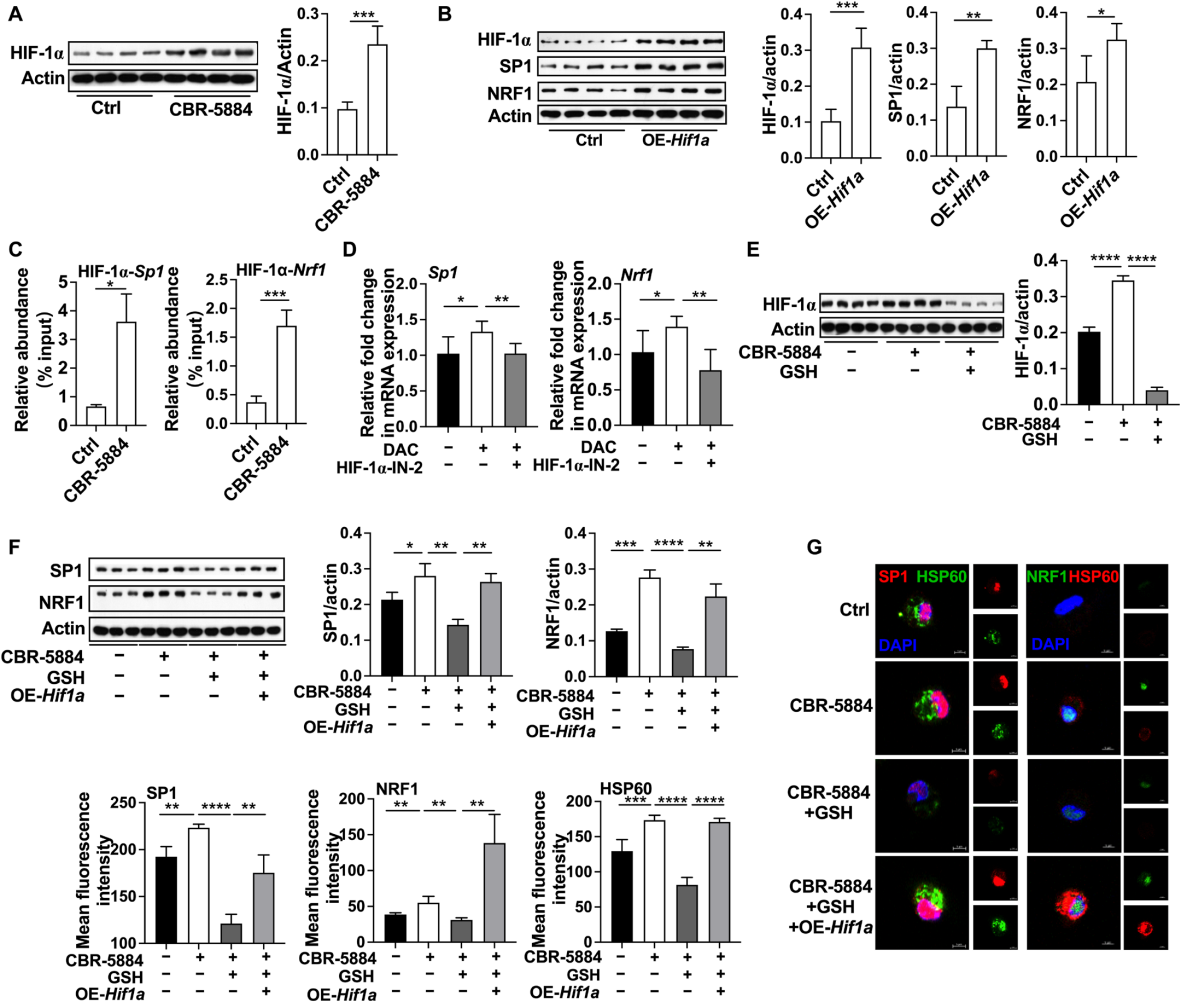
PHGDH inhibition enhances mitochondrial biogenesis through HIF-1α. (**A**) Protein abundance of HIF-1α in inflammatory macrophages treated with or without CBR-5884 (*n* = 4). (**B**) Protein abundance of HIF-1α, SP1 and NRF1 in inflammatory macrophages treated with or without *Hif1a* overexpression (*n* = 4). (**C**) ChIP-qPCR analysis of the HIF-1α occupancy at the promoter of *Sp1* and *Nrf1* in inflammatory macrophages treated with or without CBR-5884 (*n* = 5 to 7). (**D**) Relative mRNA expression of *Sp1* and *Nrf1* in inflammatory macrophages treated as indicated (*n* = 5 to 6) (HIF-1α–IN-2, 1 μM). (**E** and **F**) Protein abundance of HIF-1α, SP1 and NRF1 in inflammatory macrophages treated as indicated (*n* = 3 or 4). (**G**) Confocal microscopy of HSP60, SP1 and NRF1 in inflammatory macrophages treated as indicated (*n* = 3 to 6). Scale bar, 5 μm. The treatment time of all experiments was 12 hours. Data were analyzed by unpaired *t* test [(A) to (G)]. (A) and (B) as well as (D) to (G) are represented as means ± SD; (C) is represented as means ± SEM. *P < 0.05, **P < 0.01, ***P < 0.001, and ****P < 0.0001.

The next question is how mtROS affects SP1/NRF1 expression, thus ultimately orchestrating mitochondrial biogenesis in inflammatory macrophages. HIF-1α is a key regulator for cellular adaptation to low oxygen, and mtROS is essential for transcription and stabilization of HIF-1α (*33*). PHGDH inhibition increased the expression of HIF-1α in inflammatory macrophages (Fig. 4A and fig. S4A). Then, we overexpressed *Hif1a* in inflammatory macrophages (Fig. 4B and fig. S4B) and demonstrated that *Hif1a* overexpression promoted mRNA and protein expression of SP1 and NRF1 (Fig. 4B and fig. S4C). The binding sites of HIF-1α to *Sp1* or *Nrf1* promoters in different species were conserved (fig. S4D). Notably, our CHIP-qPCR analysis also demonstrated that PHGDH inhibition enhanced HIF-1α recruitment to *Sp1* or *Nrf1* promoter region (Fig. 4C). Thus, these data imply that HIF-1α is responsible for positive modulation of *Sp1* and *Nrf1* transcription in inflammatory macrophages with PHGDH inhibition. This hypothesis was also demonstrated with following evidence: (i) HIF-1α blockage with HIF-1α–IN-2 (*34*) inhibited DCA-induced mRNA expression of SP1 and NRF1 in inflammatory macrophages (Fig. 4D); (ii) GSH inhibited PHGDH inhibition–induced HIF-1α expression (Fig. 4E and fig. S4E); (iii) although GSH alleviated PHGDH inhibition–induced expression of SP1 and NRF1 as well as mitochondrial biogenesis, *Hif1a* overexpression blocked these effects of GSH (Fig. 4, F and G, and fig. S4F). Collectively, PHGDH inhibition enhances mitochondrial biogenesis through HIF-1α.

### PHGDH deficiency promotes mitochondrial biogenesis

**Fig. 5.**
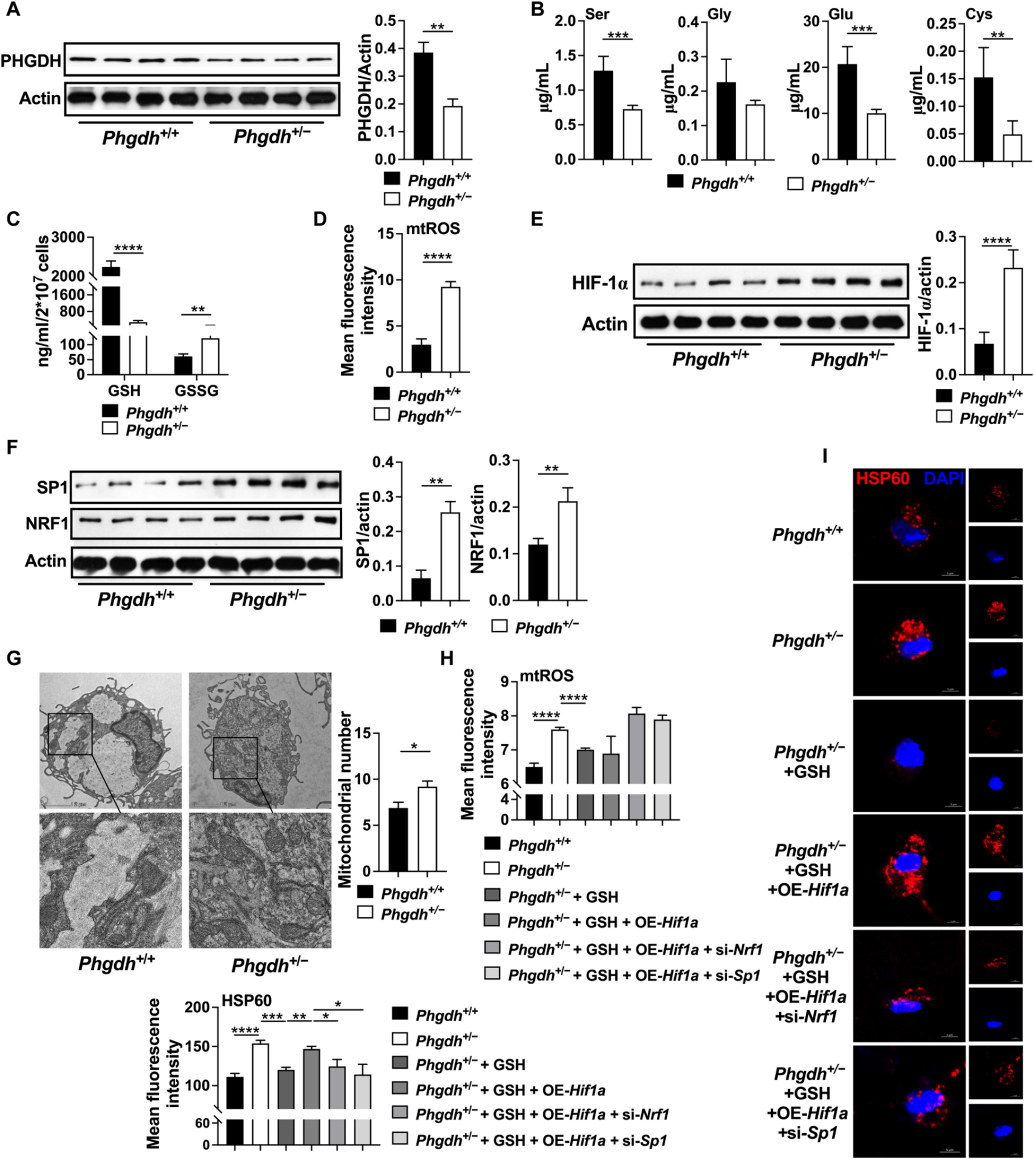
PHGDH deficiency promotes mitochondrial biogenesis. (**A**) Protein abundance of PHGDH in inflammatory macrophages from *Phgdh*^+/+^ or *Phgdh*^+/−^ mice (*n* = 4). (**B** and **C**) The glutamic acid, serine, glycine, cysteine, GSH, and GSSG level in inflammatory macrophages from *Phgdh*^+/+^ or *Phgdh*^+/−^ mice (*n* = 5). (**D**) The mtROS level in inflammatory macrophages from *Phgdh*^+/+^ or *Phgdh*^+/−^ mice (*n* = 18). (**E** and **F**) Protein abundance of HIF-1α, SP1 and NRF1 in inflammatory macrophages from *Phgdh*^+/+^ or *Phgdh*^+/−^ mice (*n* = 4). (**G**) TEM observation of mitochondrial number in inflammatory macrophages from *Phgdh*^+/+^ or *Phgdh*^+/−^ mice (*n* = 20 or 25). Scale bar, 2 μm. (**H** and **I**) The mtROS level and confocal microscopy of HSP60 (red) in inflammatory macrophages from *Phgdh*^+/+^ or *Phgdh*^+/−^ mice treated as indicated (*n* = 5). Scale bar, 5 μm. The treatment time of all experiments was 12 hours. Data were analyzed by unpaired *t* test [(A) to (I)]. (A) and (B) as well as (D) and (E) are represented as means ± SD. (C) and (F) to (I) are represented as means ± SEM. *P < 0.05, **P < 0.01, ***P < 0.001, and ****P < 0.0001.

To circumvent potential off-target effects or compensatory mechanisms associated with PHGDH inhibition, we established *Phgdh*^+/−^ mice to delete *Phgdh* at gene level (Fig. 5A and fig. S5A). We observed defects in serine, glutamic acid, cysteine, and GSH synthesis (Fig. 5, B and C, and fig. S5B) but increases in mtROS production (Fig. 5D), HIF-1α, SP1, and NRF1 expression (Fig. 5, E and F, and fig. S5, C and D), as well as mitochondrial number (Fig. 5G) in inflammatory macrophages from *Phgdh*^+/−^ mice.

To confirm the mechanism that links PHGDH and mitochondrial biogenesis, we first scavenged mtROS by supplementing GSH in inflammatory macrophages from *Phgdh*^+/−^ mice. The results showed that GSH decreased mtROS production and mitochondrial number in macrophages from *Phgdh*^+/−^ mice (Fig. 5, H and I). To further verify the importance of HIF-1α–SP1/NRF1 axis in PHGDH-mediated mitochondrial biogenesis, we overexpressed *Hif1a* in macrophages from *Phgdh*^+/−^ mice treated with GSH. As expected, *Hif1a* overexpression rescued mitochondrial number with no effect on mtROS production (Fig. 5, H and I). In such context, SP1 or NRF1 signaling lowered mitochondrial number with no effect on mtROS production (Fig. 5, H and I). Together, these results suggest that loss of PHGDH promotes mitochondrial biogenesis in macrophages.

### Myeloid depletion of *Phgdh* promotes mitochondrial biogenesis

**Fig. 6.**
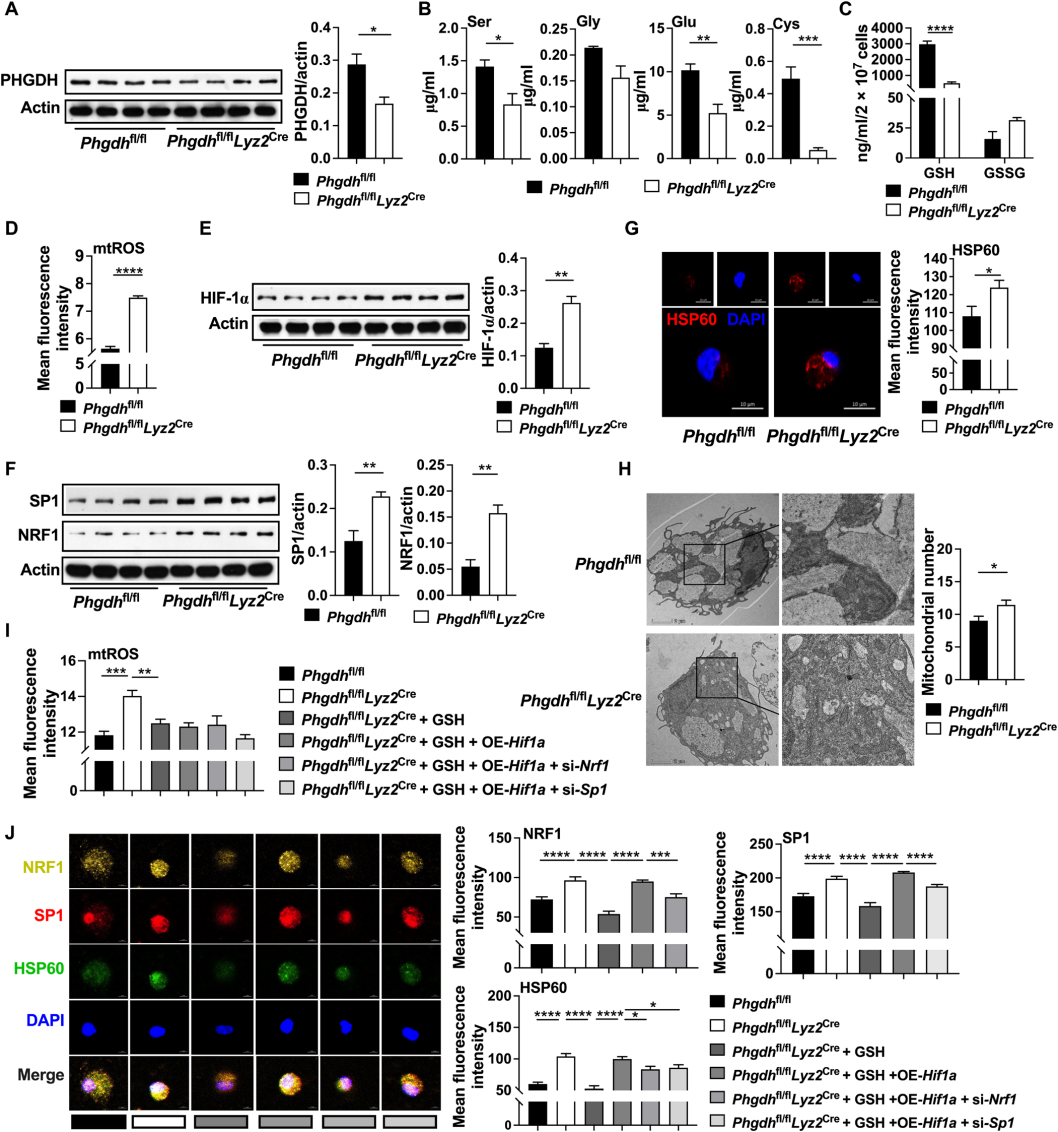
Myeloid depletion of *Phgdh* enhances mitochondrial biogenesis. (**A**) Protein abundance of PHGDH in inflammatory macrophages from *Phgdh*^fl/fl^ or *Phgdh*^fl/fl^*Lyz2*^Cre^ mice (*n* = 4). (**B** and **C**) The glutamic acid, serine, glycine, cycteine, GSH, and GSSG level in inflammatory macrophages from *Phgdh*^fl/fl^ or *Phgdh*^fl/fl^*Lyz2*^Cre^ mice (*n* = 4 or 5). (**D**) The mtROS level in inflammatory macrophages from *Phgdh*^fl/fl^ or *Phgdh*^fl/fl^*Lyz2*^Cre^ mice (*n* = 9 or 19). (**E** and **F**) Protein abundance of HIF-1α, SP1, and NRF1 in inflammatory macrophages from *Phgdh*^fl/fl^ or *Phgdh*^fl/fl^*Lyz2*^Cre^ mice (*n* = 4). (**G** and **H**) Confocal microscopy of HSP60 (red) and TEM observation of mitochondrial number in inflammatory macrophages from *Phgdh*^fl/fl^ or *Phgdh*^fl/fl^*Lyz2*^Cre^ mice (*n* = 4 to 34). Scale bar, 2 or 10 μm. (**I** and **J**) The mtROS level and confocal microscopy of NRF1 (yellow), SP1 (red) and HSP60 (green) in inflammatory macrophages from *Phgdh*^fl/fl^ or *Phgdh*^fl/fl^*Lyz2*^Cre^ mice treated as indicated (*n* = 5 to 30). Scale bar, 5 μm. The treatment time of all experiments was 12 hours. Data were analyzed by unpaired *t* test [(A) to (J)]. (A) to (J) are represented as means ± SEM. *P < 0.05, **P < 0.01, ***P < 0.001, and ****P < 0.0001.

To further confirm the intrinsic function of PHGDH in macrophages, we specifically deleted PHGDH in macrophages by establishing *Phgdh*^fl/fl^*Lyz*2^Cre^ mice (*19*) (Fig. 6A and fig. S6A). Notably, inflammatory macrophages from *Phgdh*^fl/fl^*Lyz*2^Cre^ mice had lower level of serine, glutamic acid, cysteine, and GSH (Fig. 6, B and C, and fig. S6B) compared to those from *Phgdh*^fl/fl^ mice, which is consistent with our previous study (*19*). PHGDH depletion increased mtROS production (Fig. 6D), HIF-1α, SP1, and NRF1 expression (Fig. 6, E and F, and fig. S6, C and D), as well as mitochondrial number (Fig. 6, G and H) in inflammatory macrophages.

To further verify PHGDH deficiency affects mitochondrial biogenesis through mtROS. We first supplemented GSH to inflammatory macrophages from *Phgdh*^fl/fl^*Lyz*2^Cre^ mice (fig. S6E) and found that GSH decreased mtROS production (Fig. 6I and fig. S6F), NRF1 and SP1 expression (Fig. 6J), as well as mitochondrial number (Fig. 6J and fig. S6G). NAC also decreased mtROS production and mitochondrial number in inflammatory macrophages from *Phgdh*^fl/fl^*Lyz2*^Cre^ mice (fig. S6, F and G). To further verify the importance of HIF-1α–SP1/NRF1 axis in PHGDH-mediated mitochondrial biogenesis, we overexpressed *Hif1a* in myeloid *Phgdh* deficiency inflammatory macrophages after GSH treatment. *Hif1a* overexpression rescued SP1 and NRF1 expression, as well as mitochondrial number with no effect on mtROS production (Fig. 6, I and J). Notably, in this context, SP1 or NRF1 silencing lowered mitochondrial number with no effect on mtROS production (Fig. 6, I and J). In addition, myeloid *Phgdh* deficiency failed to affect mitochondrial number in inflammatory macrophages with SP1 or NRF1 silencing (fig. S6, H and I). Collectively, these results suggest that PHGDH myeloid deficiency promotes mitochondrial biogenesis in inflammatory macrophages.

### Myeloid PHGDH deficiency reverses diet-induced obesity

**Fig. 7.**
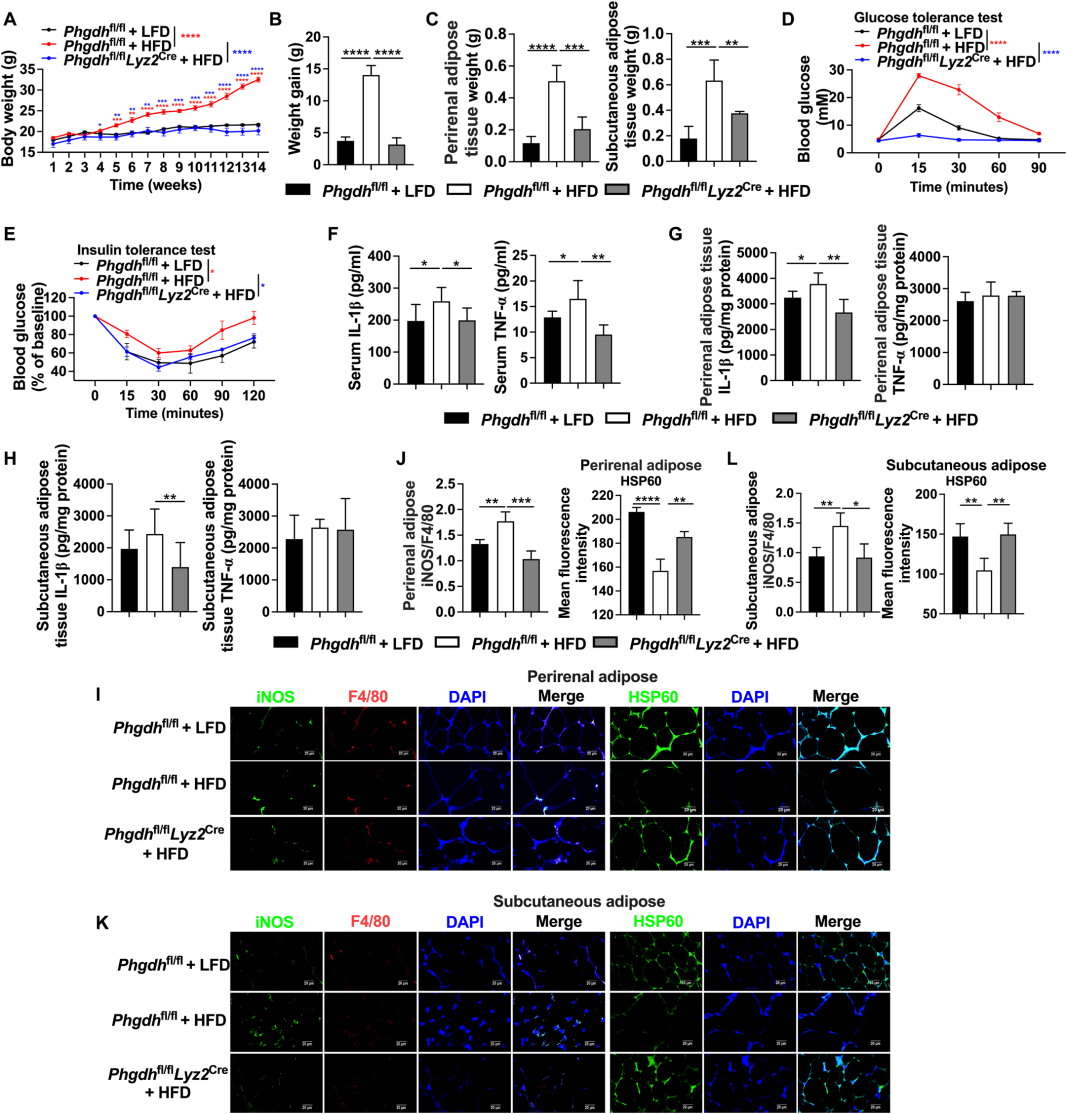
Myeloid PHGDH deficiency reverses diet-induced obesity. (**A** and **B**) The body weight and weight gain of *Phgdh*^fl/fl^ or *Phgdh*^fl/fl^*Lyz2*^Cre^ mice treated with low-fat diet (LFD) or HFD (*n* = 5 or 6). (**C**) Weight of perirenal adipose tissue and subcutaneous adipose tissue (*n* = 5 or 6). (**D** and **E**) Glucose tolerance and insulin tolerance test (*n* = 5 or 6). (**F** to **H**) IL-1β and TNF-α levels in the serum, perirenal adipose tissue and subcutaneous adipose tissue (*n* = 4 to 6). (**I** to **L**) Confocal microscopy of iNOS, F4/80 and HSP60 in perirenal adipose and subcutaneous adipose (*n* = 4). Scale bar, 20 μm. Data were analyzed by unpaired *t* test [(A) to (L)]. (A) and (C) to (G) are represented as means ± SEM; (B) and (H) to (L) are represented as means ± SD. *P < 0.05, **P < 0.01, ***P < 0.001, and ****P < 0.0001.

Inflammatory macrophage activation plays a decisive role in adipose tissue inflammation (*35*, *36*), and targeting mitochondria in macrophages holds promise for therapeutic treatment of obesity-related disorders (*37*). To verify whether PHGDH also controls mitochondrial biogenesis in adipose tissue–derived macrophages, we analyzed the mitochondrial number in adipose tissue–derived macrophages treated with PHGDH inhibitor. PHGDH inhibitor promoted mitochondrial biosynthesis in inflammatory macrophages from diet-induced obese mice (fig. S7A). To uncover whether macrophage PHGDH affects obesity via mitochondrial biosynthesis, we fed *Phgdh*^fl/fl^*Lyz2*^Cre^ mice with high-fat diet (HFD) to induce diet-induced obesity (fig. S7B). Myeloid PHGDH deficiency had no effect on average daily food intake and energy expenditure (6 weeks) (fig. S7, C to E). Notably, the body weight and body weight gain (14 weeks) were reduced in *Phgdh*^fl/fl^*Lyz2*^Cre^ mice (Fig. 7, A and B). Myeloid PHGDH deficiency also lowered weight of perirenal adipose tissue and subcutaneous adipose tissue (Fig. 7C). In addition, *Phgdh*^fl/fl^*Lyz2*^Cre^ mice showed more tolerance of glucose and more sensitivity of insulin than *Phgdh*^fl/fl^ mice (Fig. 7, D and E). Consistent with above results, *Phgdh*^fl/fl^*Lyz2*^Cre^ mice showed a lower level of IL-1β and TNF-α in the serum (Fig. 7F). Similarly, *Phgdh*^fl/fl^*Lyz2*^Cre^ mice also had a lower IL-1β level in the perirenal adipose tissue and subcutaneous adipose tissue (Fig. 7, G and H). Myeloid PHGDH deficiency lowered the ratio of inducible nitric oxide synthase (iNOS): mouse epidermal growth factor–like module-containing mucin-like hormone receptor-like 1 (F4/80) in the perirenal adipose tissue and subcutaneous adipose tissue, indicating a lower number of inflammatory macrophages in these tissues (Fig. 7, I and J). Mitochondrial number was increased in macrophages from perirenal adipose tissue and subcutaneous adipose tissue of HFD-treated *Phgdh*^fl/fl^*Lyz2*^Cre^ mice (Fig. 7, K and L). In summary, these results indicate that myeloid PHGDH deficiency reverses diet-induced obesity.

## DISCUSSION

PHGDH functions as an important regulator to activate polarization of inflammatory macrophages and thus promote inflammatory responses (*16*, *17*). Our previous study demonstrates that PHGDH promotes IL-1β production through facilitating *Tlr4* transcription and activating Leucine rich repeat and pyrin domain containing 3 inflammasome (*19*). Here, we have revealed a mechanism involved in mitochondrial biogenesis. Mitochondria serve as an important organelle that determines the fate and function of immune cells (e.g., B cells, T cells, and macrophages) (*33*, *38*–*40*). PHGDH is not only presented in the cytoplasm but also located within mitochondria, which may play an indispensable role in mitochondrial functions across various cell types (*38*, *41*). Although PHGDH deficiency has been shown to increase the mitochondrial number in endothelial cells (*18*), well-designed investigation is required to elucidate the specific role of PHGDH in the mitochondrial function of immune cells. In this study, PHGDH deficiency also promotes mitochondrial biogenesis in inflammatory macrophages. These results suggest that the regulation of PHGDH on mitochondrial biogenesis may be consistent in different cells. However, whether PHGDH regulates the function of other immune cells through mitochondrial biogenesis remains to be further studied.

Serine metabolism is recently shown to regulate inflammatory macrophage functions, which can be attributed to the activation of inflammatory signals through one carbon metabolism or nicotinamide adenine dinucleotide (oxidized form) (*16*, *19*). PHGDH serves as the rate-limiting enzyme in de novo serine synthesis, and its deficiency hampers inflammatory macrophage polarization by restricting GSH synthesis (*29*). In addition, the previous study also showed that PHGDH inhibition reduced the GSH, and GSH rescued IL-1β secretion from inflammatory macrophages (*19*). In this study, we found that PHGDH deficiency-induced GSH restriction promotes mitochondrial biogenesis. GSH synthesized in cytoplasm is transported to mitochondria through mitochondrial GSH transporter (SLC25A39) to scavenge mtROS (*42*). Unfortunately, the mitochondrial GSH in inflammatory macrophages treated with PHGDH inhibitor is not detected in our study. How mitochondria sense the abundance of cytoplasmic GSH remains to be uncovered.

The progress of mitochondrial biogenesis requires the collaborative interaction between nucleus and mitochondria (*43*). PGC-1 and PRC-1 interact with multiple transcription factors to regulate mitochondrial biogenesis (*44*–*47*). In our study, PHGDH deficiency promotes mitochondrial biogenesis thorough SP1/NRF1; however, PHGDH inhibition did not enhance the PGC-1 and PRC-1 expression. The degradation of PGC-1 and PRC-1 may be due to the increase of mtROS caused by PHGDH blockage (*48*). mtROS plays distinct roles in mitochondrial biogenesis across disparate cellular states. mtROS destroys the mitochondrial structure to release mtDNA to stimulate inflammatory macrophage polarization (*49*, *50*). However, PHGDH deletion–induced GSH synthesis deficiency may lead to macrophages being in an anoxic state, which could increase the release of mtROS and promote mitochondrial biogenesis through an mtROS–HIF-1α–SP1/NRF1 axis. These results indicate that the promotion of mitochondrial biogenesis by mtROS may depend on the degree of hypoxia. Notably, previous studies has reported that HIF-1 inhibits PGC-1α–mediated mitochondrial biogenesis by repression of myelocytomatosis viral oncogene homolog (C-MYC) activity in von hippel-lindau (VHL)–deficient renal cell carcinoma (*51*) or through basic-helix-loop-helix transcription factor Dec1 in VHL-deficient clear-cell renal cell carcinoma (*52*). The possible explanation for this difference may be related to cell types, cell microenvironments, and even the downstream regulatory mechanism of HIF-1α. Similar to mitochondrial biogenesis, the regulatory effect of HIF-1α on IL-1β production varies on the basis of the cell microenvironments and the downstream regulatory mechanism used.

Obesity arises from an imbalance between energy intake and energy expenditure (*53*, *54*). According to our data, the lean phenotype observed in myeloid PHGDH deficiency mice is unlikely to be attributable to decreased food intake or increased energy expenditure. Thus, one possibility could be that myeloid PHGDH deficiency inhibited lipid absorption in the gut when the mice were fed HFD. In addition, the adipose tissues consist of lipid-storing adipocytes, stromal cells, and immune cells (*55*). Landmark studies have firmly established that macrophages promote the storage of lipid droplets in two ways: (i) Macrophages promote the storage of lipid droplets in white adipose tissue by secreting platelet-derived growth factors (PDGFs); (ii) these white adipose tissue–resident macrophages inhibit the heat production of brown adipose tissue (*56*, *57*). Moreover, macrophages also determine the differentiation and hypertrophy of preadipocytes by cytokines (such as IL-1β and PDGFs) and other pathways (*58*). Therefore, myeloid PHGDH deficiency may mediate weight gain by the above reasons. Chronic tissue inflammation is a well-described feature of obesity, type 2 diabetes mellitus, and other insulin-resistant states (*36*). The dominant immune cells causing inflammation in obese and type 2 diabetes are macrophages (*35*). As to our current understanding, regulating macrophage polarization and secretion of inflammatory factors is an important strategy to treat obesity and type 2 diabetes mellitus. Previous studies have shown that dietary serine deficiency reduces inflammatory responses via limiting IL-1β production (*29*, *59*). De novo serine synthesis deficiency also inhibits IL-1β production and alleviates lipopolysaccharide (LPS)–induced sepsis (*19*). Here, we further demonstrated that myeloid *Phgdh* deficiency reverses diet-induced obesity. Therefore, serine metabolism, whether exogenous or de novo synthesis, is responsible for modulating IL-1β production. However, because of the tissue specificity of macrophages and complexity of diseases (*37*), whether serine metabolism is a therapeutic target for other inflammatory diseases remains to be studied.

**Fig. 8.**
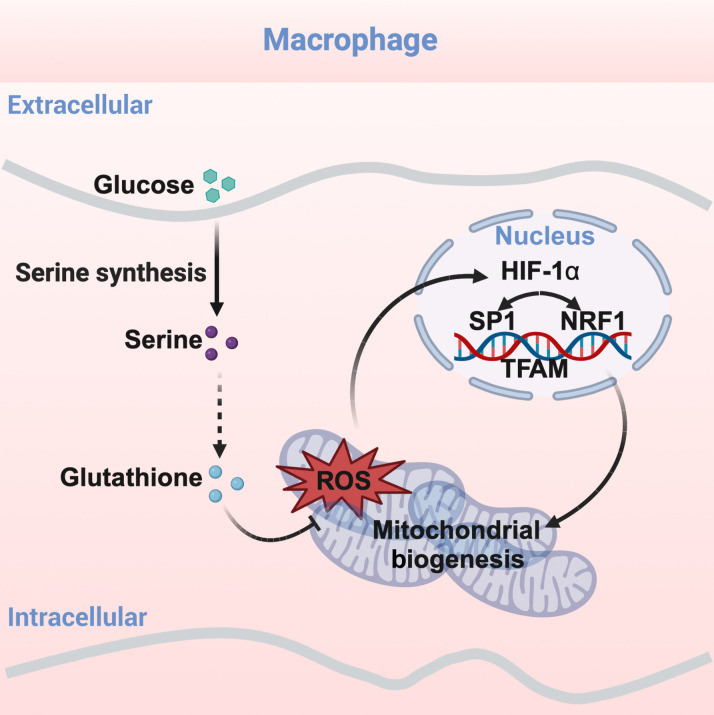
de novo serine synthesis controls mitochondrial biogenesis via mitochondrial-to-nuclear communication in inflammatory macrophages. Mechanistically, deficiency of de novo serine synthesis boosts mtROS by suppressing cytoplasmic GSH synthesis. mtROS provokes HIF-1α signaling to direct nuclear SP1 and NRF1 transcription.

Collectively, this study reveals a mechanism involving de novo serine synthesis orchestrates mitochondrial biogenesis via mitochondrial-to-nuclear communication (Fig. 8) and provides a potential therapeutic target for tackling inflammatory diseases.

## MATERIALS AND METHODS

### Cell lines

ANA-1 cells were cultured in Dulbecco’s modified Eagle’s medium (DMEM) with 1% penicillin/streptomycin and 10% fetal bovine serum and kept in a 5% carbon dioxide (CO_2_) incubator at 37°C. Cells were polarized to inflammatory macrophages with LPS (1 μg/ml) and interferon-γ (IFN-γ) (20 ng/ml) for 12 hours.

### Primary PEMs

Female 6- to 8-week-old mice (ICR or C57BL/6 J) were euthanized after 3 to 4 days of intraperitoneal injection of 2 ml of 4% sterile thioglycolate. PEMs were flushed from the peritoneal cavity with phosphate-buffered saline (PBS) and then cultured in complete DMEM. Nonadherent cells were removed after 2 to 4 hours. PEMs were polarized to inflammatory macrophages by LPS (1 μg/ml) and IFN-γ (20 ng/ml) stimulation for 12 hours except special mention.

### Adipose tissue–derived macrophages

Twenty-two-week-old female C57BL/6 J obese mice were disinfected with 75% ethanol. Adipose tissue near gonads was separated and washed with PBS to remove any contaminants before cutting into small pieces on ice. Collagenase was used to digest adipose tissue at 37°C for 20 to 45 min. Cell precipitates were filtered by PBS prewetting 100 μM nylon filter. Adipocytes and macrophages were separated by centrifugation at 500*g* for 10 min.

### Mice

Animal studies were conducted according to the guidelines of Guangdong Province on the Review of Welfare and Ethics of Laboratory Animals and approved by the South China Agricultural University Animal Care and Use Ethics Committee (2024F193). The wild-type female 6- to 8-week-old mice (C57BL/6 J and ICR) were purchased from Guangdong Sja Biotechnology Co., Ltd. (Guangzhou, China). X. Zhou (Institute of Subtropical Agriculture, Chinese Academy of Sciences, Changsha 410125, China) provided *Phgdh*^fl/fl^ (C57BL/6 J) and *Phgdh*^+/−^ (C57BL/6 J) mice. *Phgdh*^fl/fl^ *Lyz2*^Cre^ mice were derived from hybridization between Lyz2-Cre mice (The Jackson Laboratory) and *Phgdh*^fl/fl^ mice. *Phgdh*^+/+^ and *Phgdh*^+/−^ mice were obtained by crossing C57BL/6 J wild-type mice and *Phgdh*^+/−^ mice. The PCR primers used for genotyping are listed in table S2. All experiments used female 6- to 8-week-old mice with same age and gender for individual experiments. All experimental protocols were approved by the Laboratory Animal Ethics Committee of South China Agricultural University.

### HFD-induced obesity model

Animal studies were conducted according to the guidelines of Guangdong Province on the Review of Welfare and Ethics of Laboratory Animals and approved by the South China Agricultural University Animal Care and Use Ethics Committee. The female *Phgdh*^fl/fl^ and *Phgdh*^fl/fl^*Lyz2*^Cre^ mice were fed with normal diet until they were 8 weeks old. The mice were completely randomized to different experimental groups based on body weight. Then, they were maintained on HFD (60% cal of fat) or low-fat diet (6% cal of fat) for 16 weeks. The number of samples for each animal trial was specified in the corresponding legend. At 6 weeks of age, average daily feed intake and energy expenditure were measured. At 22 weeks of age, mice were injected intraperitoneally with glucose (1.5 g/kg body weight) after 12 hours of fasting for glucose tolerance test. At 23 weeks of age, mice were injected with recombinant human insulin (GLPBIO) (0.75 U/kg body weight) after 6 hours of fasting for insulin tolerance test. At 24 weeks of age, the adipose tissue and serum were sampled for further analysis.

### Gene overexpression and silencing

Gene overexpression or silencing was performed by Lipofectamine 3000 (Thermo Fisher Scientific) or Lipofectamine RNAiMAX (Thermo Fisher Scientific) transfection reagent according to the manufacturer’s protocol. The plasmid and siRNA oligonucleotides are listed in table S2.

### Real-time quantitative polymerase chain reaction

Total RNA was extracted by RNA Purification Kit (EZBioscience) on the basis of the manufacturer’s instructions. RNA was reverse transcribed to cDNA by the Reverse Transcription Master Mix (EZBioscience). Reverse transcription qPCR was conducted by the Quant Studio 6 PCR System (Thermo Fisher Scientific) and 2 × Color SYBR Green qPCR Master Mix (EZBioscience). The primers are listed in table S2. Fold change was assessed using the 2^−ΔΔCt^ method using β-*actin* as a reference gene.

### Western blot

The total protein of inflammatory macrophages were washed with PBS and lysed in radioimmunoprecipitation assay buffer with protease inhibitors (Beyotime). After that, the protein concentration was detected by bicinchoninic acid protein assay kit (Beyotime). Then, the same amount of protein was detected by SDS–polyacrylamide gel electrophoresis and transferred to a poly(vinylidene fluoride) membrane (BioRad). Membranes were blocked with 5% bovine serum albumin tris-Tween–buffered saline buffer for 2 hours at room temperature. After blocking, the membranes were incubated with primary and secondary antibodies and visualized using chemiluminescent reagent sequentially. The antibodies we sued were listed in table S1. Last, the developed blots were observed and analyzed by a light imaging system (Bio-Rad) and ImageJ software.

### Immunofluorescence

For cell samples, the inflammatory macrophages after the appropriate treatments were fixed with 4% paraformaldehyde for 20 min at room temperature. The samples were blocked with Quickblock blocking buffer (Beyotime) at room temperature for 30 min. The primary antibodies incubated with the fixed and blocked cells at 4°C for 12 hours. Then, the cells were incubated with the secondary antibodies at 37°C for 1.5 hours. The nucleus was stained with 4′,6-diamidino-2-phenylindole (DAPI) (Beyotime) at room temperature for 10 min. Stained cells were observed using a confocal fluorescence microscope (Zeiss), and data were analyzed by ImageJ or ZEN 3.2.

For tissues samples, the sections were deparaffinized, rehydrated, antigen repaired, and washed in 0.5% Tween 20 phosphate buffer solution (PBST), treated with 3% Triton X-100, and blocked with Quickblock blocking buffer (Beyotime). Other steps were the same as cell samples.

### Transmission electron microscopy

The preparation of transmission electron microscopy samples according to the standard protocol. Macrophages were fixed in 2.5% glutaraldehyde for 24 hours and 1% osmium tetroxide at 4°C for 4 hours. Samples were negatively stained with uranyl acetate and lead citrate, dehydrated with gradient ethanol, and embedded in epoxy resin according to the procedure. The embedded cells were sliced by a 60-nm ultra-microtome (EM UC7, Leica) and observed by transmission electron microscope (Talos L120C, FEI). The mitochondrial area was analyzed by ImageJ.

### Chromatin immunoprecipitation–quantitative polymerase chain reaction

ChIP assays (Cell Signaling Technology) were performed according to the protocols provided by the manufacturer. Chromatin was digested to 200 to 800 bp and immunoprecipitated with a specific antibody after cross-linking chromatin proteins with formaldehyde (Sigma-Aldrich) and then separated and purified the DNA for ChIP-qPCR.

### Amino acid and GSH/GSSG assays

The levels of amino acids, GSH and GSSG in lysed cells and mitochondria were analyzed by a liquid chromatograph mass spectrometer according to the standard protocol.

### mtROS assays

Macrophages were washed with Hanks’ balanced salt solution and incubated with 5 μM MitoSox (Thermo Fisher Scientific) at 37°C for 30 min. Fluorescence intensity was obtained by confocal fluorescence microscopy.

### Cytokines assays

The cytokine levels in serum and tissue were detected using the enzyme-linked immunosorbent assay kits (Proteintech) according to the manufacturer’s protocol.

### Statistical analysis

Results represent an independent experiment unless indicated. Statistical analysis methods and biological replicates of each experiment were indicated in the corresponding figure legends. For the blinding to histological analysis and immunofluorescence, samples from each group were named after neutral codes and then given to investigators not involved in the experimental design for quantification. All data were analyzed by GraphPad Prism 9.0 and presented as means ± SEM or SD. Data were analyzed by unpaired *t* test if they were Gaussian distributed with equal variances, by Welch-corrected unpaired *t* test if they were Gaussian distributed with unequal variances, or by Mann-Whitney *U* tests if they were not normally distributed. Differences with *P* < 0.05 were considered significant.
