# Supplementary material for: Serine synthesis controls mitochondrial biogenesis in macrophages
Source: Sci Adv. 2024 May 17;10(20):eadn2867. doi: 10.1126/sciadv.adn2867 (PMC11100566; doi:10.1126/sciadv.adn2867)
Supplement: Supplementary file 1 — Figs. S1 to S7 Tables S1 and S2 [file sciadv.adn2867_sm.pdf]

Science Advances
AAAS

Supplementary Materials for

Serine synthesis controls mitochondrial biogenesis in macrophages

Chuanlong Wang *et al.*

Corresponding author: Wenkai Ren, renwenkai19@scau.edu.cn

Sci. Adv. **10**, eadn2867 (2024)
DOI: 10.1126/sciadv.adn2867

This PDF file includes:

Figs. S1 to S7
Tables S1 and S2

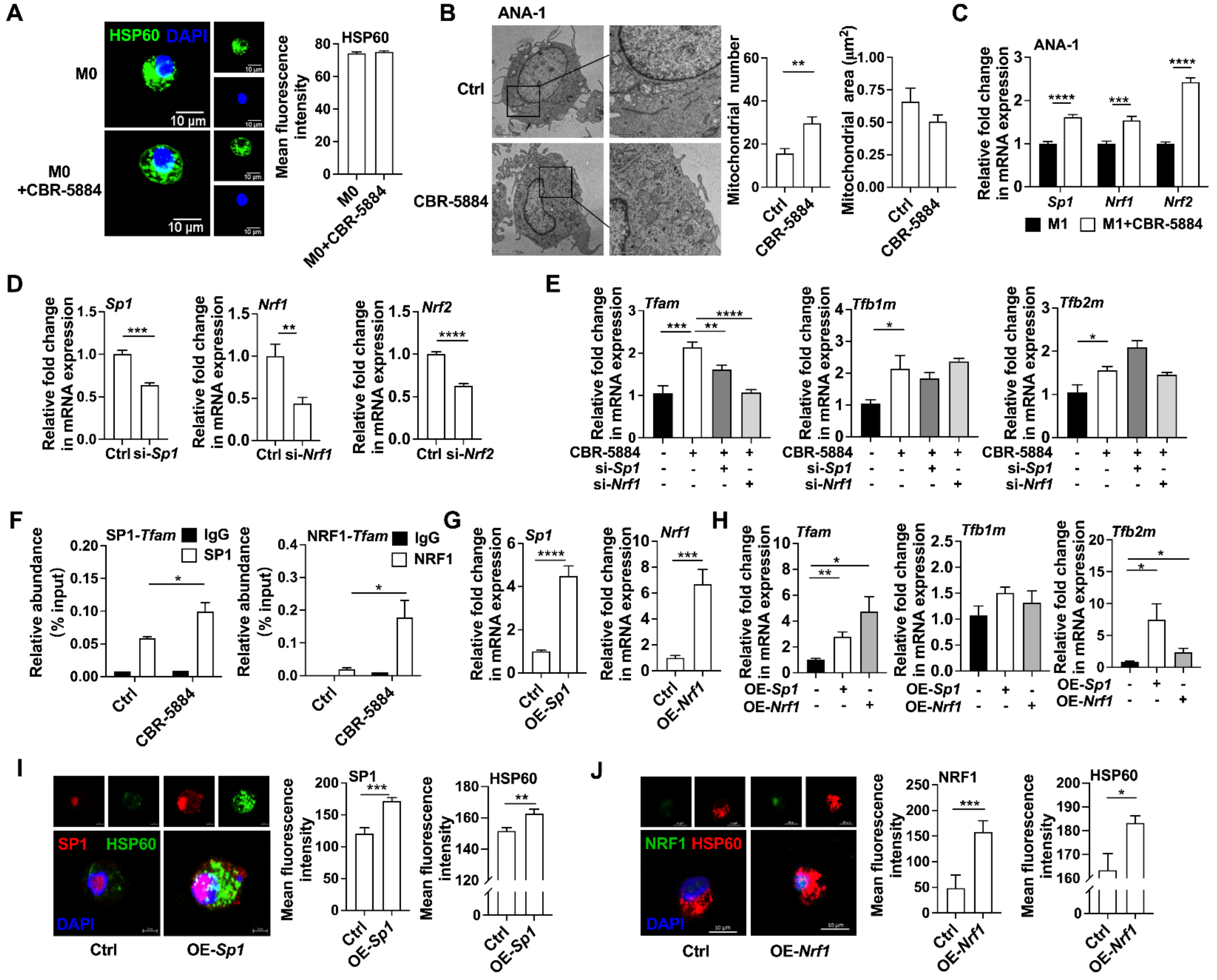

Figure S1. PHGDH inhibition promotes mitochondrial biogenesis. (**A**) Confocal microscopy of HSP60 (red) in quiescent macrophages treated with or without CBR-5884 (30 μM) (n = 6). Scale bar as 10 μm. (**B**) Transmission electron microscopy (TEM) observation for mitochondrial number and area in ANA-1 treated with or without CBR-5884. Scale bar as 2 μm. (**C**) Relative mRNA expression of *Sp1*, *Nrf1* and *Nrf2* in ANA-1 treated with or without CBR-5884 (n = 6). (**D-E and G-H**) Relative mRNA expression of *Sp1*, *Nrf1*, *Nrf2*, *Tfam*, *Tfb1m* and *Tfb2m* in inflammatory macrophages treated as indicated (n = 4-6). (**F**) ChIP-qPCR analysis of the SP1/NRF1 occupancy at the promoter of *Tfam* in inflammatory macrophages treated with or without CBR-5884 (n = 5). (I-J) Confocal microscopy of HSP60, SP1 and NRF1 in inflammatory macrophages treated as indicated (n=6). Scale bar as 5 or 10 μm. The treatment time of all experiments was 12 h. Data were analyzed by unpaired *t* test (A, B left and C-J) or Mann-Whitney U test (B right). A-J represented as means ± SEM. *P<0.05, **P<0.01, ***P<0.001 and ****P<0.0001.

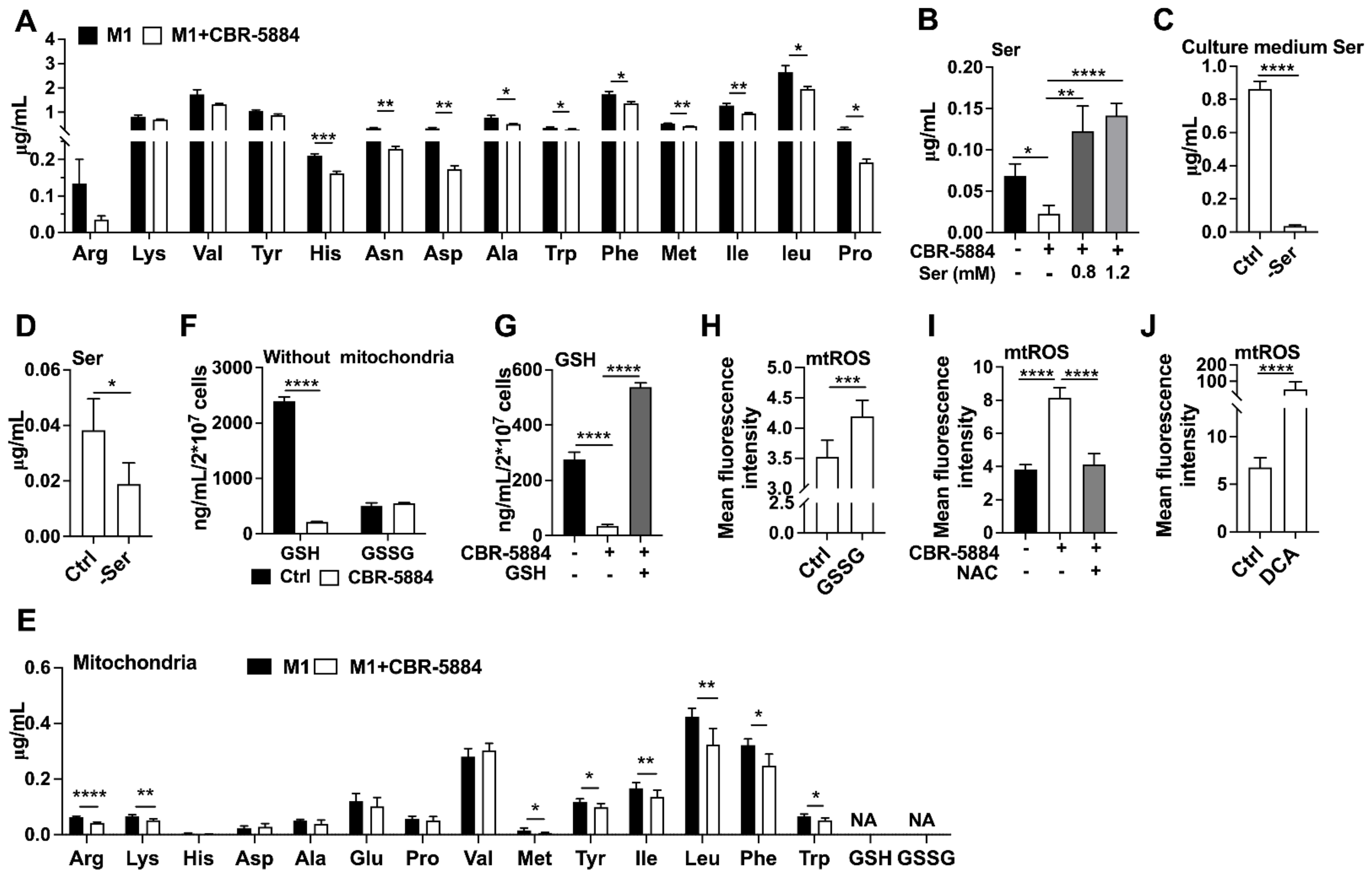

Figure S2. PHGDH inhibition enhances mitochondrial biogenesis through mtROS. (**A**) Intracellular amino acids in inflammatory macrophages treated with or without CBR-5884 (n = 6). (**B-D**) Serine concentration in inflammatory macrophages or culture medium treated as indicated (n = 4). (**E-G**) Amino acids, GSH and GSSH in inflammatory macrophages treated with or without CBR-5884 (n = 4 or 6). (**H-J**) The mtROS level in inflammatory macrophages treated as indicated (n = 9). The treatment time of all experiments was 12 h. Data were analyzed by unpaired *t* test (A-G and I-J) or Mann-Whitney U test (H). A and F represented as means ± SEM; B-E and G-J represented as means ± SD. **P*<0.05, ***P*<0.01, ****P*<0.001 and *****P*<0.0001.

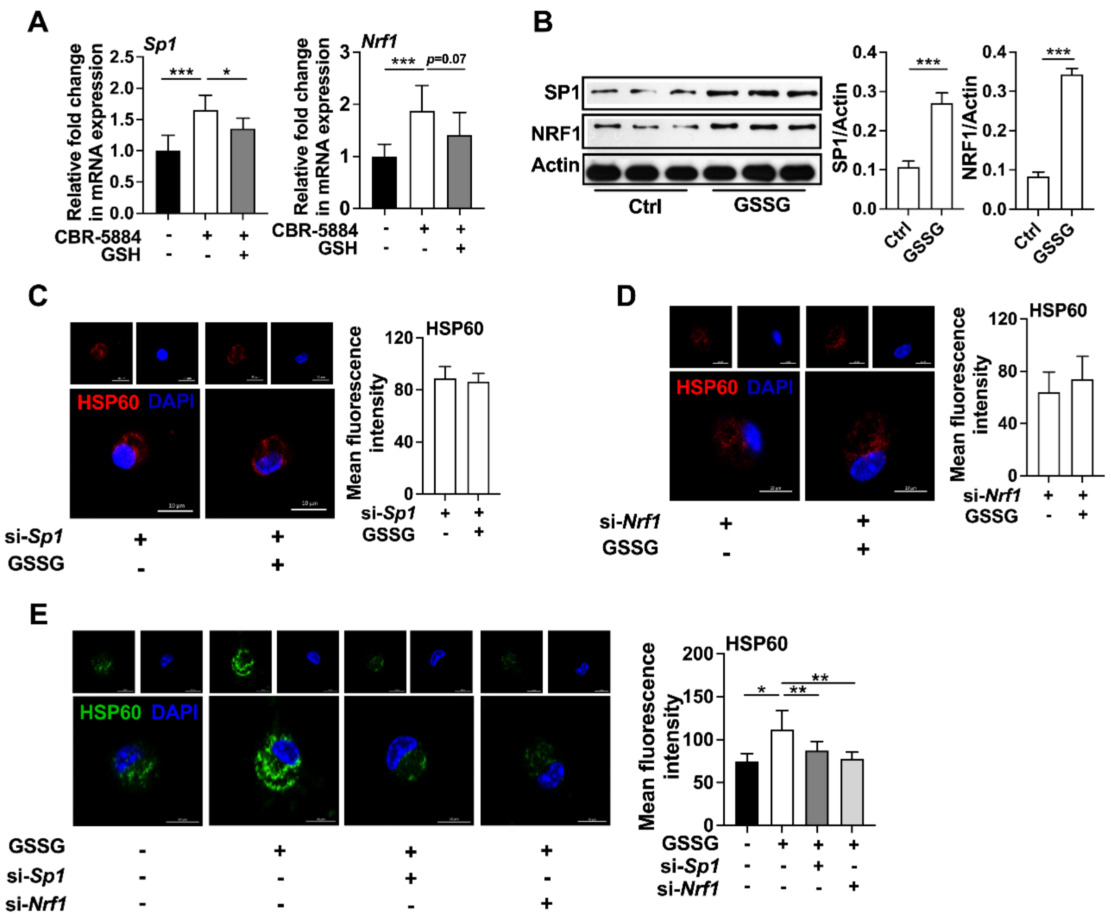

Figure S3. mtROS promotes mitochondrial biogenesis *via* SP1/NRF1. (**A**) Relative mRNA expression of *Sp1* and *Nrf1* in inflammatory macrophages treated as indicated (GSH, 1.25 mM) (n = 6-8). (**B**) Protein abundance of SP1and NRF1 in inflammatory macrophages treated with or without GSSG (GSSG, 1.25 mM) (n = 3). (**C-E**) Confocal microscopy of HSP60 (red) in inflammatory macrophages treated as indicated (n = 4-10). Scale bar as 10 μm. The treatment time of all experiments was 12 h. Data were analyzed by unpaired *t* test and represented as means ± SD. *P<0.05, **P<0.01 and ***P<0.001.

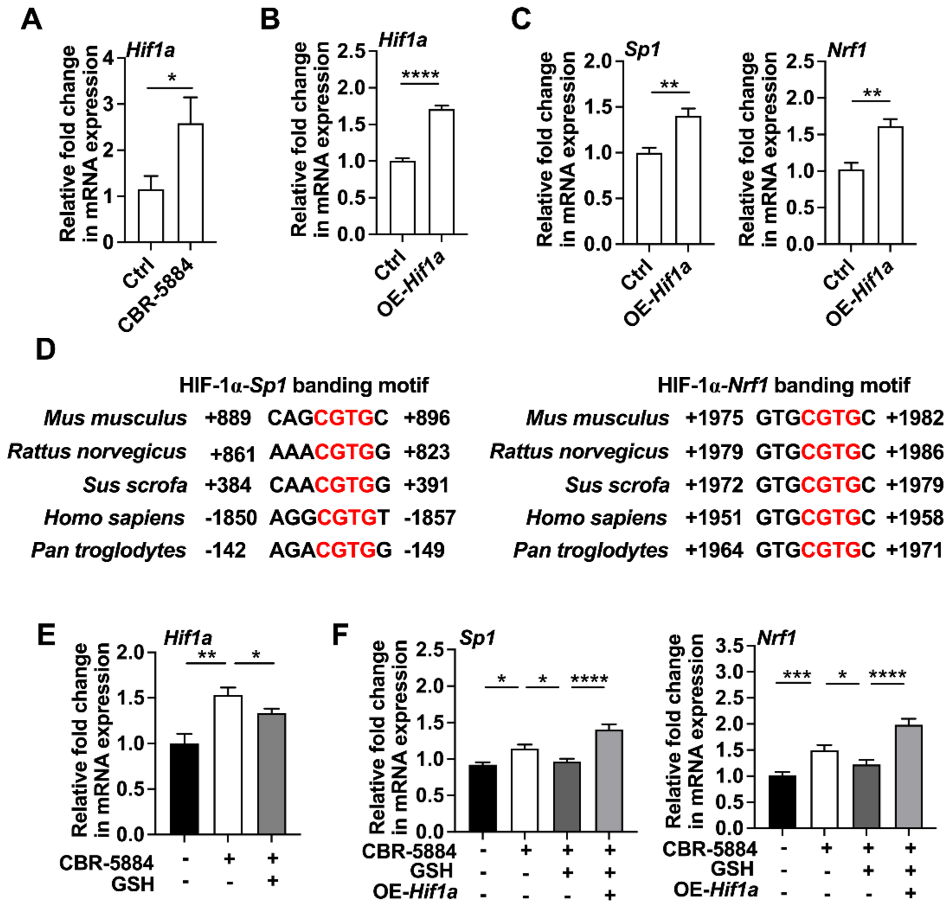

Figure S4. PHGDH inhibition enhances mitochondrial biogenesis through HIF-1α. (**A-C**) Relative mRNA expression of *Hif1a, Sp1* and *Nrf1* in inflammatory macrophages treated with or without CBR-5884 or *Hif1a* overexpression (n = 6 or 8). (**D**) The binding sites of HIF-1α with *Sp1* and *Nrf1* promoters in different species. (**E-F**) Relative mRNA expression of *Hif1a, Sp1* and *Nrf1* in inflammatory macrophages treated with or without CBR-5884 or *Hif1a* overexpression (n = 6-18). The treatment time of all experiments was 12 h. Data were analyzed by unpaired *t* test and represented as means ± SEM. $^*P<0.05$, $^{**}P<0.01$, $^{***}P<0.001$ and $^{****}P<0.0001$.

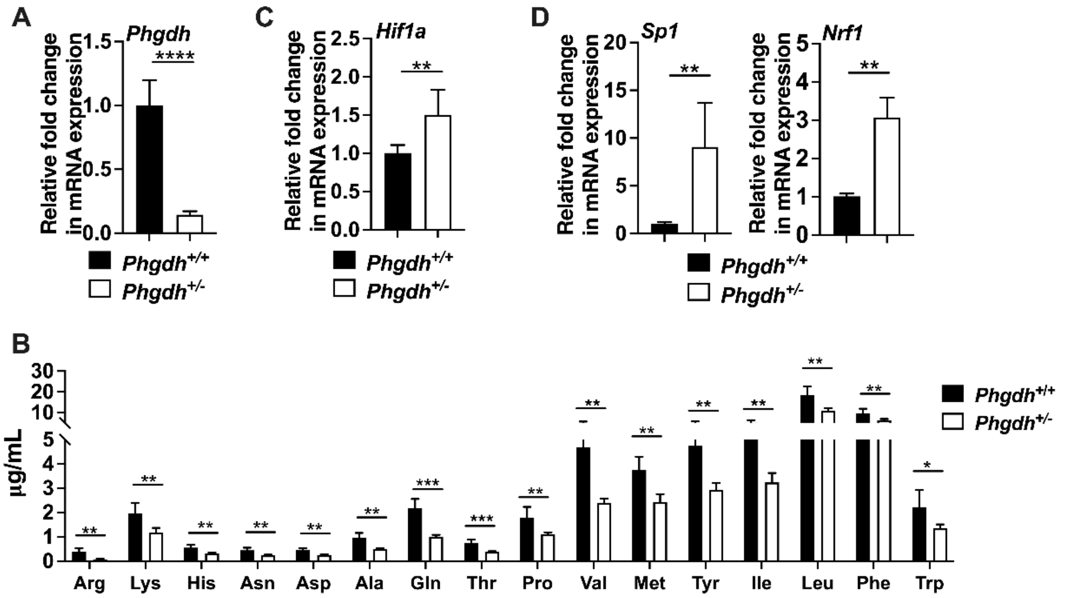

Figure S5. PHGDH deficiency promotes mitochondrial biogenesis. (**A-D**) Relative mRNA expression of *Phgdh, Hif1a, Sp1* and *Nrf1* in inflammatory macrophages from *Phgdh*$^{+/+}$ or *Phgdh*$^{+/-}$ mice (n = 6). Intracellular amino acids concentration in inflammatory macrophages from *Phgdh*$^{+/+}$ or *Phgdh*$^{+/-}$ mice (n = 5). The treatment time of all experiments was 12 h. Data were analyzed by unpaired *t* test and represented as means ± SD. *P<0.05, **P<0.01, ***P<0.001 and ****P<0.0001.

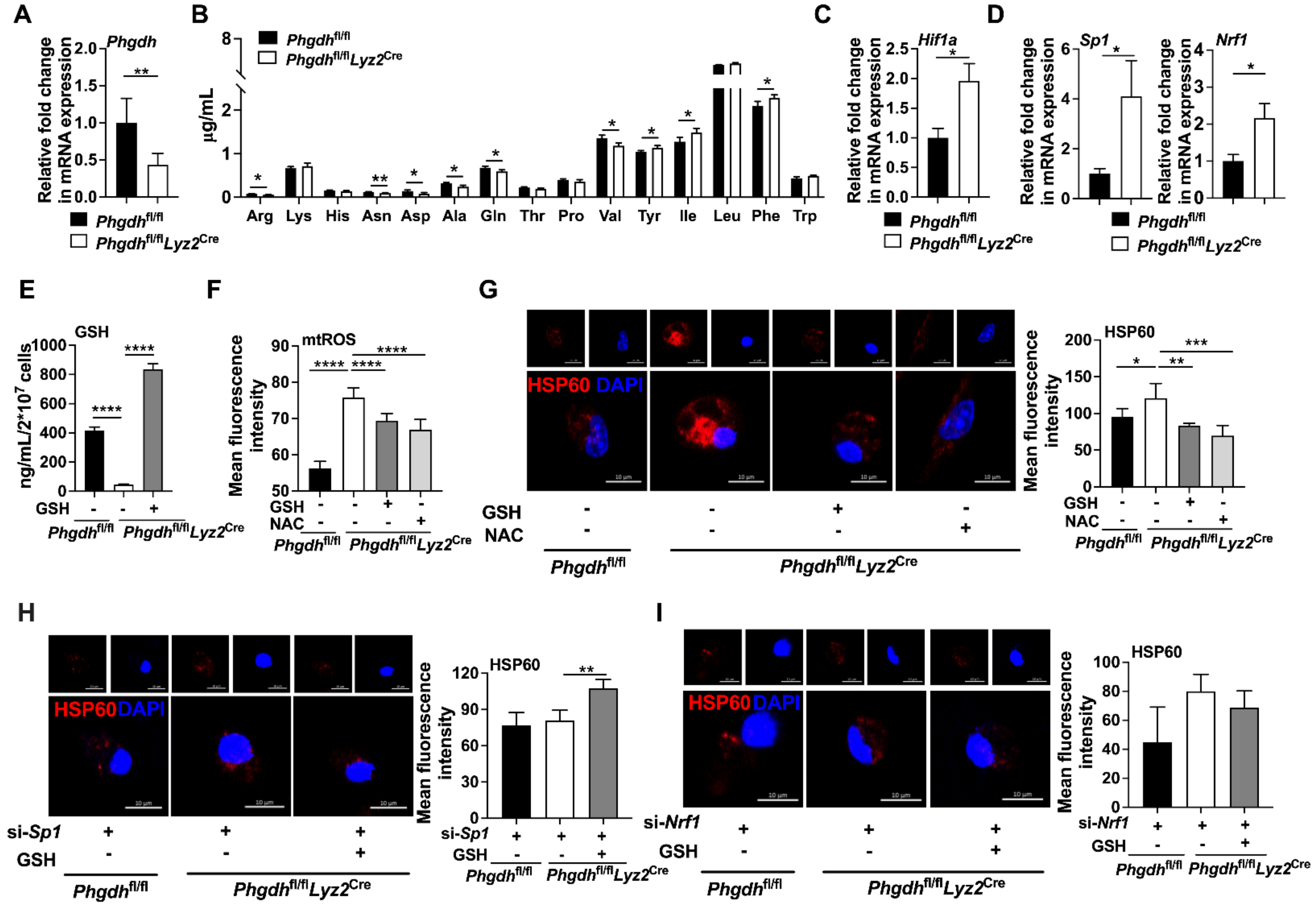

Figure S6. Myeloid depletion of *Phgdh* enhances mitochondrial biogenesis. (**A and C-D**) Relative mRNA expression of *Phgdh, Hif1a, Sp1* and *Nrf1* in inflammatory macrophages from *Phgdh*$^{fl/fl}$ or *Phgdh*$^{fl/fl}$*Lyz2*^{Cre} mice (n = 7-11). (**B and E**) Intracellular amino acids concentration and GSH in inflammatory macrophages from *Phgdh*$^{fl/fl}$ or *Phgdh*$^{fl/fl}$*Lyz2*^{Cre} mice treated as indicated (n = 4). (**F**) The mtROS level in inflammatory macrophages from *Phgdh*$^{fl/fl}$ or *Phgdh*$^{fl/fl}$*Lyz2*^{Cre} mice treated as indicated (GSH and NAC, 1.25 mM) (n = 8-22). (**G-I**) Confocal microscopy of HSP60 in inflammatory macrophages from *Phgdh*$^{fl/fl}$ or *Phgdh*$^{fl/fl}$*Lyz2*^{Cre} mice treated as indicated (n = 4-8). Scale bar as 10 μm. The treatment time of all experiments was 12 h. Data were analyzed by unpaired *t* test (A-I). A-D and G-I represented as means ± SD. E-F represented as means ± SEM. **P*<0.05, ***P*<0.01, ****P*<0.001 and *****P*<0.0001.

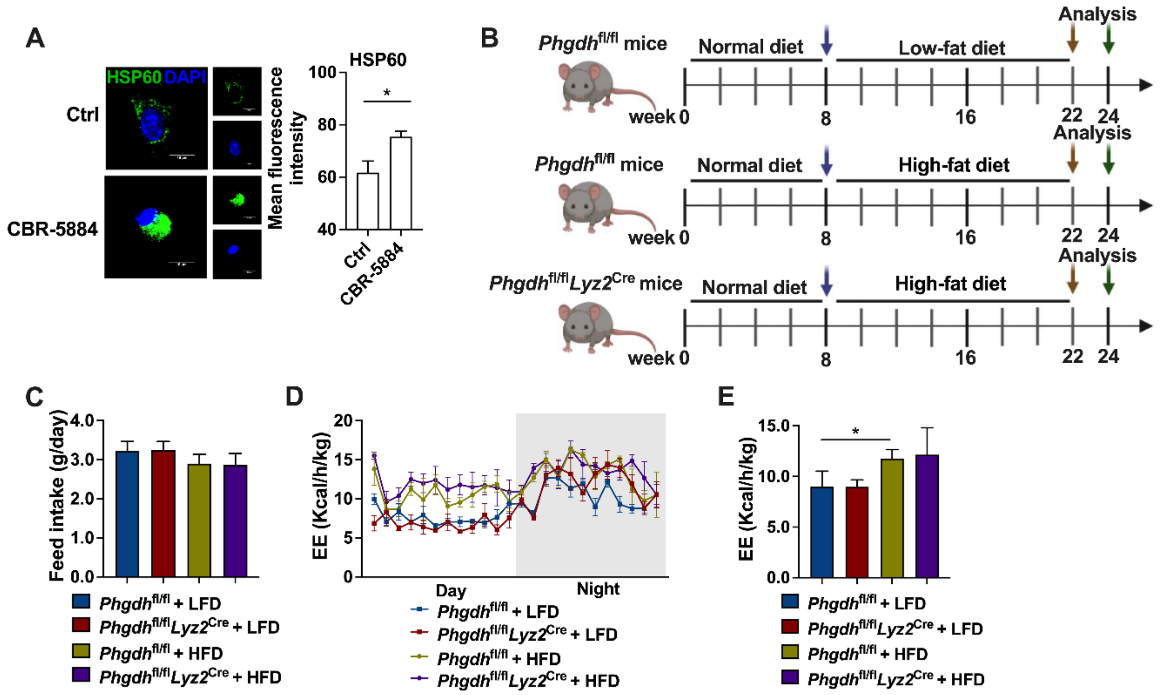

Figure S7. Myeloid PHGDH deficiency reverses diet-induced obesity. (**A**) Confocal microscopy of HSP60 in adipose tissue-derived macrophages treated with or without CBR-5884 (n = 5 or 7). Scale bar as 10 μm. (**B**) Schematic diagram of experimental plans for diet-induced obesity (16 weeks) (n=8). (**C-E**) Food intake and energy expenditure of *Phgdh*$^{fl/fl}$ and *Phgdh*$^{fl/fl}$*Lyz2*^{Cre} mice feed with high-fat or low-fat diet diet (6 weeks) (n=4). Data were analyzed by unpaired *t* test (A, C and E). A represented as means ± SEM. C and E represented as means ± SD. **P*<0.05.

Table S1. Key resources table.

**Reagent**	**Sourcce**	**Identifier**
**Antibodies**		
PGC-1α	Abclonal	Cat#A12348
PRC-1	Proteintech	Cat#CL488-67027;<br>RRID: AB_2919409
SP1	Proteintech	Cat#21962-1-AP;<br>RRID: AB_10898171
NRF1	Proteintech	Cat#12482-1-AP；<br>RRID: AB_ 2282876
NRF2	Proteintech	Cat#16396-1-AP;<br>RRID: AB_2782956
BNIP3L	Abcam	Cat#ab155010
HIF-1α	Proteintech	Cat#20960-1-AP;<br>RRID: AB_10732601
PHGDH	Proteintech	Cat#14719-1-AP;<br>RRID: AB_2283938
β-actin	Proteintech	Cat#66009-1-Ig;<br>RRID: AB_2687938
HSP60	Proteintech	Cat#66041-1-Ig;<br>RRID: AB_11041709
SP1	Proteintech	Cat#21962-1-AP;<br>RRID: AB_10898171
HIF-1α	Novus	Cat#NB100-105;<br>RRID: AB_1643856
F4/80	Santa Cruz	Cat#sc-377009;<br>RRID: AB_2927461
iNOS	Abclonal	Cat#A3774
**Chemicals**		
CBR-5884	APExBIO	Cat#A8721
DAPI	Beyotime	Cat#C1006
Sodium dichloroacetate	MCE	Cat#HY-Y0445A
NAC	Beyotime	Cat#ST-1546
GSH	Solarbio	Cat#G8180
GSSH	Solarbio	Cat#G8690
HIF-1α-IN-2	MCE	Cat#HY-115903
Lipopolysaccharide (LPS)	Sigma Aldrich	Cat#L2630

Recombinant murine IFN-γ	PeproTech	Cat#315-05
Lipofectamine™ 3000	Invitrogen	Cat#L3000015
**Critical commercial assays**		
IL-1β ELISA	Proteintech	Cat#KE10003
TNF-α ELISA	Proteintech	Cat#KE10002
mtROS Kit	Thermo Fisher	Cat#M36008
SimpleChIP® Enzymatic Chromatin IP Kit (Agarose Beads)	CST	Cat#9002S
FuGENE® HD Transfection reagent	Promega	Cat#E2311
Reduced Glutathione (GSH) Content Assay Kit	Solarbio	Cat#BC1175
**Cell lines**		
ANA-1	Yangzhou University	RRID: CVCL_0142
**Software**		
Image J	National Institutes of Health	https://www.imagej.net/
ZEN 3.2	Zeiss	https://www.zeiss.com/
Graphpad Prism 9	GraphPad Software	https://www.graphpad.com/
QuantStudioTM Real-Time PCR V1.7.2	QuantStudio™ Real-Time PCR Software	Applied biosystems by Thermo Fisher

Table S2. Oligonucleotides, primers and recombinant DNA

Reagent	Source
**Oligonucleotides**	
siRNA for *Sp1* : GCCCTTATTACCACCAATA	Ribobio
siRNA for *Nrf1*: CACATTGGCTGATGCTTCA	Ribobio
siRNA for *Nrf2*: CAGCCACGCTGAAAGTTCA	Ribobio
**Primers for qPCR**	
Pgc-1 FW (CGATGACCCTCCTCACACCAAAC)	Sangon Biotech
Pgc-1 RV (CGATGACCCTCCTCACACCAAAC)	Sangon Biotech
Prc-1 FW (ACGGCAAACGGAAGCAGAAGAC)	Sangon Biotech
Prc-1 RV (GGGCTGATGGAACAAGGCAAGG)	Sangon Biotech
Sp1 FW (GAAGCAGCAGCACAGGCAGTAG)	Sangon Biotech
Sp1 RV (GCCAGCAGAGCCAAAGGAGATG)	Sangon Biotech
Nrf1 FW (GCTACTTACACCGAGCATAGTA)	Sangon Biotech
Nrf1 RV (CTCAAATACATGAGGCCGTTTC)	Sangon Biotech
Nrf2 FW (CAGCCATGACTGATTTAAGCAG)	Sangon Biotech
Nrf2 RV (CAGCTGCTTGTTTTCGGTATTA)	Sangon Biotech
Atg7 FW (GTGTACGATCCCTGTAACCTAG)	Sangon Biotech
Atg7 RV (GATGCTATGTGTCACGTCTCTA)	Sangon Biotech
Bcl2l13 FW (CCTGTGGTCTGTAGTGTTGTTGGTG)	Sangon Biotech
Bcl2l13 RV (CCGCCTTCATATCGCTGCTTGG)	Sangon Biotech
Fkbp8 FW (GAAGGTCAAGTGTCTGAACAAC)	Sangon Biotech
Fkbp8 RV (TTGGGATGGCCTCACTATATTC)	Sangon Biotech
Bnip3l FW (GGCAGATCATGTTTGATGTTGA)	Sangon Biotech
Bnip3l RV (CCAGTCTGCACTTTTCTTCAAA)	Sangon Biotech
Ulk1 FW (ACTCAGGTGCACAATTACCAG)	Sangon Biotech
Ulk1 RV (CTTGGGGAGAAGGTGTGTAG)	Sangon Biotech
Lc3 FW (CTGTCCTGGATAAGACCAAGTT)	Sangon Biotech
Lc3 RV (GTCTTCATCCTTCTCCTGTTCA)	Sangon Biotech
Pink1 FW (CATCGCCTATGAAATCTTTGGG)	Sangon Biotech
Pink1 RV (AATTTCAGGTTCTTCAGGGCTA)	Sangon Biotech
Ndp52 FW (TGCTATGTGGATGAAGATGGTT)	Sangon Biotech

Ndp52 RV (GCTGTTCCATCTCTTCTACCTT)	Sangon Biotech
Usp8 FW (GAAGAAATGGGGAGAATTGTGC)	Sangon Biotech
Usp8 RV (CATATGAACTGTGTTTGTGGGG)	Sangon Biotech
Hif1a FW (GAATGAAGTGCACCCTAACAAG)	Sangon Biotech
Hif1a RV (GAGGAATGGGTTCACAAATCAG)	Sangon Biotech
Phgdh FW (CCTCATTGTCCGGTCTGCTAC)	Sangon Biotech
Phgdh RV (CATCTTTCATCGAAGCTGTTGC)	Sangon Biotech
Tfam FW (ACCGTATTGCGTGAGACGAA)	Sangon Biotech
Tfam RV (TGAAAGTTTTGCATCTGGGTGT)	Sangon Biotech
Tfb1m FW (TACAGGAAGCAAACAGCACAGTCG)	Sangon Biotech
Tfb1m RV (TGGGCTCTATCAAGGGCGTAAGG)	Sangon Biotech
Tfb2m FW (AGCAAGAATGACGCCACAACAGG)	Sangon Biotech
Tfb2m RV (GCTCGGGGTCTTTCGCTTTCG)	Sangon Biotech
**Primers for genotype**	
Phgdh KO P1 (CAAAAATAGGTAGGACAGGTGGTG)	Sangon Biotech
Phgdh KO P2 (GCTGGGATAGTGAGGGAAATAGAG)	Sangon Biotech
Phgdh KO P3 (GGCTGGTGCTGCTTATAGGAGA)	Sangon Biotech
Phgdh KO P4 (GGCTGGGACAGTGAGAACATTG)	Sangon Biotech
Phgdh flox P1 (GGCTGGTGCTTATAGGAGA)	Sangon Biotech
Phgdh flox P2 (GGCTGGGACAGTGAGAACATTG)	Sangon Biotech
Mutant reverse (CCCAGAAATGCCAGATTACG)	Sangon Biotech
Lyz2-cre common (CTTGGGCTGCCAGAATTTCTC)	Sangon Biotech
**Primers for ChIP-qPCR**	
Wild type reverse (TTACAGTCGGCCAGGCTGAC)	Sangon Biotech
Sp1 FW (AGCGTGCGTTACTCTGAAAGC)	Sangon Biotech
Sp1 RV (TTTCGGGAAGATGTAGCAAGCG)	Sangon Biotech
Nrf1 FW (GTAGACCAGACTAGCCTCGAACTC)	Sangon Biotech
Nrf1 RV (CACGCCTTTAATCCCAGCACTC)	Sangon Biotech
**Recombinant DNA**	
Plasmid: pcDNA3.1-*Sp1*	Gene create
Plasmid: pcDNA3.1-*Nrf1*	Gene create
Plasmid: pcDNA3.1-*Hif1a*	Gene create
